# Ovarian BDNF promotes survival, migration, and attachment of tumor precursors originated from p53 mutant fallopian tube epithelial cells

**DOI:** 10.1038/s41389-020-0243-y

**Published:** 2020-05-29

**Authors:** Min Kang, Kay Yi Chong, Tobias M. P. Hartwich, Fangfang Bi, Allyson K. Witham, David Patrick, Madeline J. Morrisson, Sarah L. Cady, Alexandra P. Cerchia, Dawn Kelk, Yifei Liu, Jonah Nucci, Oluwagbemisola Madarikan, Daiki Ueno, Brian M. Shuch, Yang Yang-Hartwich

**Affiliations:** 1 Department of Obstetrics, Gynecology, and Reproductive Sciences, Yale School of Medicine, New Haven, CT 06510 USA; 2 The First Affiliated Hospital of Guangxi Medical University, 530022 Nanning, Guangxi China; 3 Sheng Jing Hospital of China Medical University, 110004 Shenyang, Liaoning China; 4 Department of Biology and Environmental Science, University of New Haven, West Haven, CT 06516 USA; 5 Ronald Reagan UCLA Medical Center, University of California Los Angeles, Santa Monica, CA 90095 USA; 6 Yale Cancer Center, New Haven, CT 06510 USA

**Keywords:** Ovarian cancer, Growth factor signalling

## Abstract

High-grade serous ovarian carcinoma (HGSOC) is the most lethal gynecological malignancy. New evidence supports a hypothesis that HGSOC can originate from fallopian tube epithelium (FTE). It is unclear how genetic alterations and pathophysiological processes drive the progression of FTE tumor precursors into widespread HGSOCs. In this study, we uncovered that brain-derived neurotrophic factor (BDNF) in the follicular fluid stimulates the tropomyosin receptor kinase B (TrkB)-expressing FTE cells to promote their survival, migration, and attachment. Using in vitro and in vivo models, we further identified that the acquisition of common TP53 gain-of-function (GOF) mutations in FTE cells led to enhanced BDNF/TrkB signaling compared to that of FTE cells with *TP53* loss-of-function (LOF) mutations. Different mutant p53 proteins can either increase TrkB transcription or enhance TrkB endocytic recycling. Our findings have demonstrated possible interplays between genetic alterations in FTE tumor precursors (i.e., p53 GOF mutations) and pathophysiological processes (i.e., the release of follicular fluid upon ovulation) during the initiation of HGSOC from the fallopian tube. Our data revealed molecular events underlying the link between HGSOC tumorigenesis and ovulation, a physiological process that has been associated with risk factors of HGSOC.

## Introduction

High-grade serous ovarian carcinoma (HGSOC) is the most common histologic type of ovarian cancer. The majority of patients are diagnosed at advanced stages due to the lack of early detection or prevention strategies. The pathogenesis of HGSOC is not fully understood.

The historically prevalent theory on the origins of HGSOC was that HGSOC initiates from ovarian surface epithelium or cortical inclusion cysts^1–3^. New evidence suggests that HGSOCs can originate from the fallopian tube (FT) epithelium^4–9^. Specifically, serous tubal intraepithelial carcinomas (STICs) and morphologically normal cells carrying *TP53* mutation were identified as potential tumor precursors in the FT fimbriae of *BRCA1/BRCA2* mutation carriers^10–12^. These precursors coexist with advanced HGSOC and carry *TP53* mutation identical to that of the coexisting HGSOC^13–15^. In mouse models, the same mutations as those identified in human HGSOC can initiate HGSOC-like tumors from oviducts that are equivalent to human FT^16–19^. Despite these advances in understanding the origin and genomics of HGSOC, it is still unclear how genetic alterations and pathophysiological processes promote HGSOC initiation and progression.

*TP53* mutation is the most frequent mutation in HGSOC^20–22^. p53 is a central regulator for maintaining normal cellular and tissue homeostasis. Loss of wild-type p53 impairs cell-cycle checkpoint controls, protects cells from stress stimuli during oncogenic events, and facilitates malignant transformation (as reviewed in refs. ^23,24^). Mutant p53 protein can interact with new DNA targets and protein partners to promote genomic instability, invasion, metastasis, proliferation, inflammation, angiogenesis, and chemoresistance^24^. HGSOC patients with gain-of-function (GOF) p53 mutations have a worse prognosis^25^. The most frequent p53 mutations in HGSOC occur at codons R273, R248 and R175. They are all GOF mutations with frequencies of 8.31%, 6.02%, and 5.53% in all p53 mutations, respectively^26^. p53R273H promotes HGSOC through inhibiting lysophosphatidic acid phosphatase type 6 and increasing lipid secretion in fallopian tube epithelium (FTE) cells^27^. p53R248W binds to Rad21 to stimulate ovarian cancer cell invasion^28^. p53R175H upregulates fibronectin, integrin α5, and TWIST1 expression to promote cell aggregation upon the detachment of FTE cells^29^. The mouse homolog of p53R175H promotes transformation, invasion, and metastasis of epithelial ovarian cancer in mice^18,19,30^.

Tubal/ovarian microenvironment also has a profound impact on tumor precursors. FT fimbriae are in close proximity to the ovary and repeatedly exposed to follicular fluid (FF) upon ovulation. The reactive oxygen species, mitogens, growth factors (e.g. IGF and transferrin), chemoattractants (e.g. SDF-1), and hormonal components in FF have been implicated in ovarian cancer pathogenesis^31–36^. Epidemiological studies suggest the protective effects of oral contraceptive use, increased parity, and breastfeeding against ovarian cancer^37–39^. These factors are associated with reduced ovulation cycles.

This study focuses on understanding the roles of brain-derived neurotrophic factor (BDNF) and its receptor TrkB in HGSOC initiation from the FT. BDNF is highly expressed in the brain as a nerve growth factor that induces the migration, survival, and differentiation of neurons^40^. Ovarian BDNF regulates follicle development and oocyte maturation^41–44^. BDNF/TrkB signaling inhibits anoikis, the apoptosis induced by detaching from extracellular matrix (ECM), and promotes the progression of ovarian, cervical, colon, breast, lung, and gastric cancers^45–53^. TrkB overexpression is associated with large tumor size, metastases, and late-stage diseases^54^. It is a prognostic marker for ovarian cancer^55^. We have identified that fallopian tube epithelial cells (FTEs) express TrkB, which responds to the ovary-secreted BDNF to promote their survival, migration, and adhesion. Our data unveiled the interplays between genetic alterations (i.e., p53 GOF mutations) and microenvironmental factors (i.e., BDNF in ovarian FF).

## Results

### p53 mutation and detachment from ECM induce TrkB expression in FTEs

**Fig. 1:**
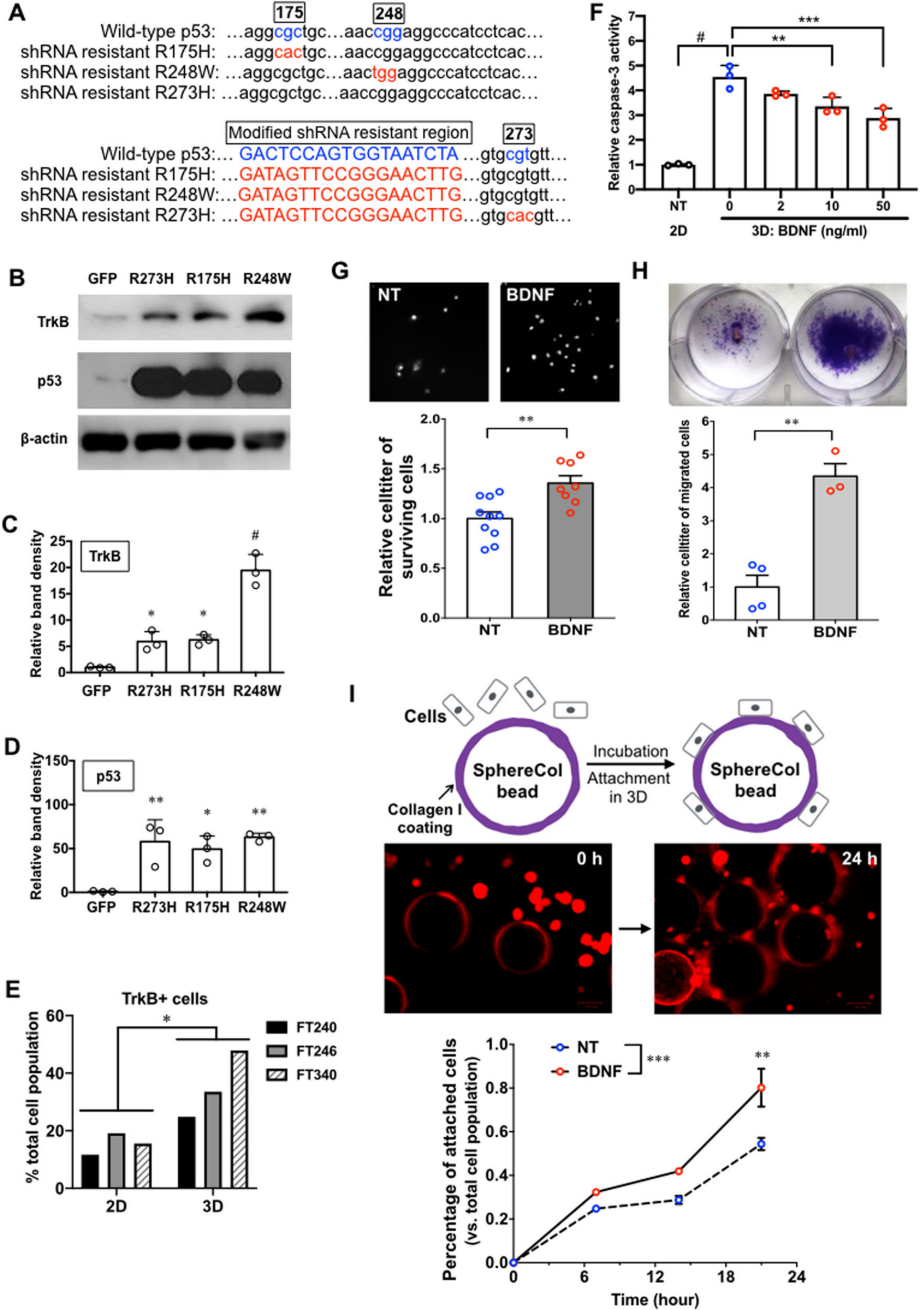
BDNF promotes the survival, migration and attachment of fallopian tube epithelial cells (FTEs). **a** Partial sequences of wild-type p53 and shRNA-resistant p53 mutants. The graph was based on the plasmid DNA sequencing result. **b** Representative images of western blot. The whole-cell lysate of FT240 cells overexpressing green fluorescent protein (GFP), p53R273H, p53R175H, or p53R248W were analyzed. **c**, **d** Band density quantification of p53 and TrkB western blot. Band density was normalized to the data of β-actin control and compared to the control FT240 expressing GFP. *n* = 3. *p < 0.05, **p < 0.005, and ^#^*p* < 0.0001 for one-way ANOVA followed by Tukey’s HSD test. **e** Flow cytometry of TrkB in human fallopian tube cell lines FT240, FT246, and FT340. 2D and 3D indicate the regular 2D adhesion culture and cell suspension 3D culture, respectively. *p < 0.05 for paired Student’s *t* test. **f** BDNF suppressed Caspase3/7 activity of FT240 cells in 3D culture. Data were normalized to Caspase3/7 activity of untreated cells in 2D culture (NT 2D). *n* = 3. **p < 0.005, ***p < 0.0005, and ^#^*p* < 0.0001 for one-way ANOVA followed by Tukey’s HSD test. **g** BDNF (50 ng/ml) promoted the recovery of FT240 cells (FTEs) from anoikis-inducing condition. Cell viability was quantified after FTEs reattached to collagen I-coated matrix for 48 h. DAPI staining was used to visualize the nuclei (as white dots) of the recovered cells in the representative images. Cell viability was determined using CellTiter-Glo 2D Cell Viability Assay. *n* = 8. **p < 0.005 for unpaired Student’s *t* test. **h** BDNF (50 ng/ml) accelerated the migration of FTEs from hydrogel. Migrated cells were visualized by crystal violet staining. After hydrogel pieces were removed, the migrated cells were quantified with CellTiter-Glo 2D Cell Viability Assay. *n* = 3. **p < 0.005 for unpaired Student’s *t* test. **i** BDNF (50 ng/ml) enhanced the attachment of FTEs to Collagen I-coated beads. Representative images indicate the attachment of red fluorescent FT240 to beads after 24-h incubation as expected. The percentage of attached cells is incubation-time-dependent. BDNF treatment accelerated the attachment. **p < 0.005 and ***p < 0.0005 for two-way ANOVA followed by Sidak HSD test.

We identified that human and mouse normal FTEs expressed TrkB (Supplementary Figs. S1 and S2). Human FTE cell lines, FT240 and FT246, were immortalized by viral transduction of human telomerase reverse transcriptase, p53 shRNA, and CDK4^R24C^^56^. In these cell lines, we overexpressed mutant p53R175H, R248W, and R273H by changing the shRNA-targeted sequence into shRNA-resistant sequence without altering the encoded amino-acid residues (Fig. 1a and Supplementary Methods). The overexpression of mutant p53 increased the levels of TrkB protein (Fig. 1b−d and Supplementary Fig. S3). When we cultured FTE cell lines FT240, FT246 and FT340 in three-dimensional (3D) condition that mimics the detachment of FTEs from ECM, they expressed higher levels of TrkB protein than that of the FTEs in 2D culture condition (Fig. 1e).

Collectively, GOF p53 mutations R175H, R248W, and R273H increased the level of TrkB in FTEs. Detachment from ECM followed by anchor-independent culture condition also enhanced TrkB expression. These observations led us to hypothesize that the pathological activation of TrkB promotes the initiation of HGSOC from fallopian tube tumor precursors.

### BDNF suppresses anoikis of FTEs

To better understand the role of BDNF/TrkB signaling in HGSOC tumorigenesis, we determined whether BDNF affected the tolerance of FTE cells to anoikis. FTE cell lines were cultured in 3D condition for 24 h to induce Anoikis, in which Caspase-3/7 activity was significantly higher than that of the adherent 2D culture. BDNF treatment suppressed their caspase-3/7 activity in a dose-dependent manner without changing cell numbers (Fig. 1f and Supplementary Figs. S4−S6). The inhibition of apoptosis by BDNF further led to increased viability of FTE cells after 48 h. We only observed this prosurvival effect of BDNF under serum-free 3D culture, not under adherent and/or full-serum conditions (Supplementary Figs. S7 and S8). Moreover, FT240 and FT246 cells that were treated by 50 ng/ml BDNF for 24 h in 3D condition recovered better than the untreated cells when they reattached to collagen I-coated plates (Fig. 1g and Supplementary Fig. S9). Since BDNF was present in the 3D culture, but not during recovery, this result demonstrates the prolonged protective effects of BDNF on FTEs upon reattachment.

### BDNF stimulates the migration and adhesion of FTEs

The ability to migrate and adhere is important for tumor precursors to spread and initiate tumors. When FTEs were seeded inside a piece of hydrogel, they migrated outside onto the petri dish. Crystal violet stained migrating cell-covered areas that were significantly larger in the BDNF-treated group than that of the untreated group, which was confirmed by celltiter quantification data (Fig. 1h and Supplementary Fig. S10).

In a 3D adhesion model, we incubated FTEs with human collagen I-coated SphereCol beads. The attachment of FTEs was increased in a time-dependent manner as expected (Fig. 1i). BDNF increased the cell attachment to 80.2 ± 8.7% after 21 h comparing to that of the untreated control cells at 54.4 ± 2.8%. These data indicate that BDNF enhances the ability of FTEs to overcome anoikis, migrate, and attach to secondary sites, which can allow them to initiate tumors outside the FT.

### The ovary promotes FTEs migration and adhesion through BDNF/TrkB signaling

**Fig. 2:**
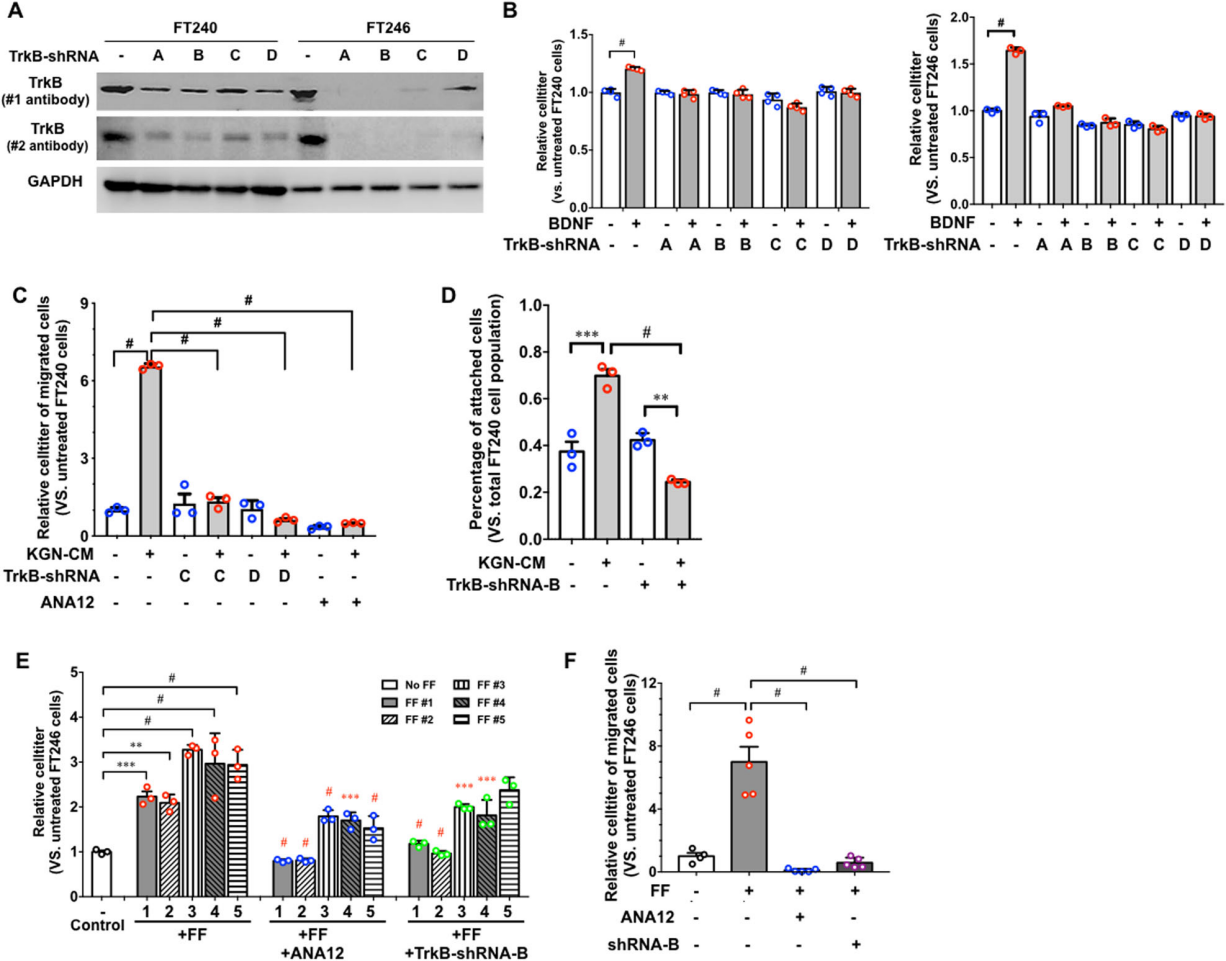
Ovary-secreted factors promote the survival, migration and attachment of FTEs through BDNF/TrkB pathway. **a** Western blot images of TrkB in two FTE cells lines, FT240 and FT246, indicate the knockdown of TrkB by shRNA-A, B, C, and D. Two antibodies (#1 and #2) were used to validate the knockdown. **b** TrkB-shRNAs inhibited the BDNF-enhanced cell viability of FT246 cells in 3D culture. FT246 expressing nontarget shRNA was used as negative control. *n* = 3. ^#^*p* < 0.0001 for two-way ANOVA followed by Sidak HSD test. **c** Granulosa cell line KGN conditional medium (CM) induced the migration of FT240 cells. TrkB-shRNAs-C and D, and a TrkB antagonist, ANA-12, inhibited the KGN-CM-induced migration. *n* = 3. ^#^*p* < 0.0001 for one-way ANOVA followed by Tukey’s HSD test. **d** KGN CM enhanced the attachment of FT240 to ECM in 3D model. TrkB-shRNA-B inhibited the KGN CM-induced attachment. *n* = 3. **p < 0.001, ***p < 0.0005, and ^#^*p* < 0.0001 for two-way ANOVA followed by Sidak HSD test. **e** Human follicular fluid (FF) increased the cell viability of FT246. ANA-12 and TrkB-shRNA-B inhibited the FF-induced increase of cell viability. Each number (1−5) indicates an FF sample from an individual woman. FT246 expressing nontarget shRNA was cultured in Opti-MEM-reduced serum medium as negative control. *n* = 3. **p < 0.001, ***p < 0.0005, and ^#^*p* < 0.0001 for one-way ANOVA followed by Tukey’s HSD test. In the ANA12- and shRNA-B-treated groups, ***p < 0.0005 and ^#^*p* < 0.0001 in red color indicate the comparisons to the matched sample treated with the FF from the same woman. **f** Five individual FF samples induced the migration of FT246 cells. ANA-12 and TrkB-shRNAs-B inhibited the FF-induced migration. *n* = 5. ^#^*p* < 0.0001 for one-way ANOVA followed by Tukey’s HSD test.

BDNF is expressed by the ovary and secreted into FF^41,42^. In order to determine the role of BDNF/TrkB in the interaction between FTEs and the ovary, we knocked down TrkB in FT240 and FT246 cells using lentiviral shRNAs and validated the knockdown by western blot using two different anti-TrkB antibodies (Fig. 2a). These shRNAs effectively inhibited the BDNF-stimulated increase of 3D cell viability in FT240 and FT246 cells (Fig. 2b).

Granulosa cells were identified as the main ovarian cells that secrete BDNF^57^. In vitro cultured granulosa cell line KGN secretes BDNF^58^. We demonstrated that KGN conditional medium (CM) promoted the migration of FT240 cells, which was inhibited by TrkB-shRNAs and ANA-12, a TrkB antagonist (Fig. 2c). KGN CM also significantly enhanced the attachment of FTEs to the collagen I-coated beads after 14-h incubation, which was inhibited by TrkB-shRNA-B (Fig. 2d).

Similarly, medium containing 50% human FF significantly enhanced the survival of FT246 cells in 3D culture compared to the control group without FF (Fig. 2e). ANA-12 and TrkB-shRNA-B inhibited the prosurvival activity of FF (Fig. 2e). Additionally, FF stimulated FT246 cell migration, and ANA-12 and TrkB-shRNA-B suppressed their migration (Fig. 2f). These results are consistent among five individual FF samples and demonstrate that FF depends on TrkB signaling to stimulate the survival and migration of FTE tumor precursors.

### Mutant p53 enhances BDNF/TrkB signaling in FTEs

FTE tumor precursors gain *TP53* mutation as an early event of tumorigenesis^10,11^. We found that three common p53 GOF mutations upregulated TrkB, indicating that the acquisition of GOF *TP53* mutation can enhance the oncogenic activity of TrkB in tumor precursors and contribute to tumor initiation. We tested this hypothesis in several models in vitro and in vivo.

**Fig. 3:**
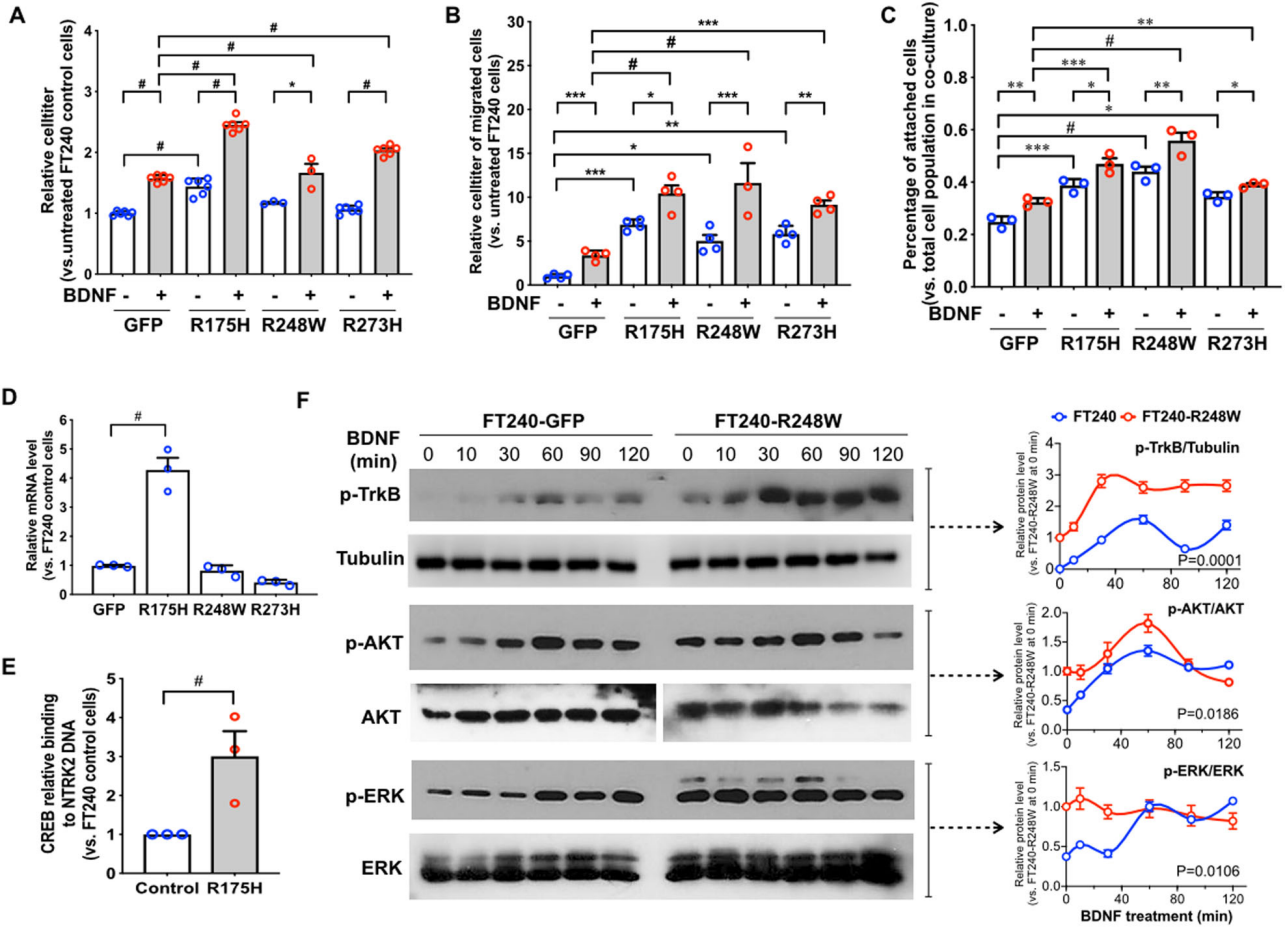
p53 mutations enhance BDNF/TrkB signaling in fallopian tube tumor precursors. **a** Mutant p53 enhanced the survival of FT240 cells stimulated by 50 ng/ml BDNF in 3D culture for 48 h. *n* = 6. **b** Mutant p53 increased the migration of FT240 cells from hydrogel stimulated by 50 ng/ml BDNF. *n* = 4. **c** Mutant p53 promoted the BDNF-stimulated (50 ng/ml) attachment of FT240 cells to Collagen I-coated beads. *n* = 3. **a**−**c** *p < 0.05, **p < 0.005, ***p < 0.0005, and ^#^*p* < 0.0001 for two-way ANOVA followed by Sidak HSD test. **d** Mutant p53-R175H, but not R248W or R273H, induced TrkB mRNA expression of FT240 cells. GAPDH was used as internal control. *n* = 3. ^#^*p* < 0.0001 for one-way ANOVA followed by Tukey’s HSD test. **e** The binding of CREB to NTRK2 DNA was increased in p53-R175H-expressing FT240 cells than control cells as assessed by chromatin-IP of CREB followed by QPCR. *n* = 3. (See Supplementary Fig. S11 for the data of each biological repeat before normalization.) ^#^*p* < 0.0001, paired Student’s *t* test. **f** The overexpression of p53-R248W in FT240 cells led to enhanced activation of TrkB, AKT, and ERK by BDNF (50 ng/ml) treatment. Relative band density of phosphorylated TrkB normalized to Tubulin (p-TrkB/Tubulin), phosphorylated AKT normalized to total AKT (p-AKT/AKT), and phosphorylated ERK normalized to total ERK (p-ERK/ERK) were quantified and normalized to the relative expression level of FT240-R248W cells at 0 min. *n* = 3. *p* values were calculated using two-way ANOVA followed by Sidak HSD test.

First, we determined that the proliferation of FTEs (Supplementary Fig. S11) and their tolerance to anoikis were enhanced by mutant p53 overexpression comparing to that of the control FTEs overexpressing GFP (Fig. 3a). In particular, p53R175H and p53R273H augmented the BDNF-induced increase of cell viability in 3D. Second, mutant p53 enhanced both the basal levels of migration and BDNF-stimulated migration of FTEs (Fig. 3b). Finally, mutant p53 significantly increased the FTE attachment to collagen I-coated beads and their BDNF-stimulated attachment (Fig. 3c). These data support the hypothesis that *TP53* GOF mutations R175H, R248W, and R273H enhance the oncogenic function of BDNF/TrkB signaling in tumor precursors.

### *TP53* mutations enhance BDNF/TrkB signaling via distinctive mechanisms

In order to understand the molecular mechanism by which GOF mutant p53 enhances BDNF/TrkB activity, we first determined the extent to which mutant p53 affected the transcription of TrkB (encoded by *NTRK2* gene). RT-QPCR result showed that p53R175H, but not R248W or R273H, significantly increased TrkB mRNA level (Fig. 3d). Therefore, different p53 mutations may exert distinctive GOF to promote BDNF/TrkB signaling.

It was reported that cAMP response element-binding protein (CREB) binds to *NTRK2* promoter to activate its transcription^59^. We demonstrated the direct interaction of p53R175H and CREB in FTEs by co-IP (Supplementary Fig. S12). p53R175H overexpression significantly increased the binding of CREB to *NTRK2* promoter as assessed by chromatin-IP (Fig. 3e and Supplementary Figs. S13 and S14).

Since p53R248W and p53R273H upregulated TrkB protein without increasing mRNA transcription, we hypothesized that post-translational regulatory pathways were involved, such as the endocytic pathway that regulates the trafficking, degradation, and recycling of TrkB^60^. Upon activation by BDNF, cell surface TrkB internalizes and degrades in lysosomes or recycled back to the surface in endosomes. As a critical component of endosomes, Golgi Associated Gamma Adaptin Ear Containing ARF Binding Protein 3 (GGA3) promotes the switch towards receptor recycling and thus sustains the activation of downstream pathways^61,62^, which may account for the increased levels of TrkB protein, leading to the prolonged activation of TrkB pathway as evidenced by the enhanced phosphorylation of TrkB, AKT, and ERK (Fig. 3f).

**Fig. 4:**
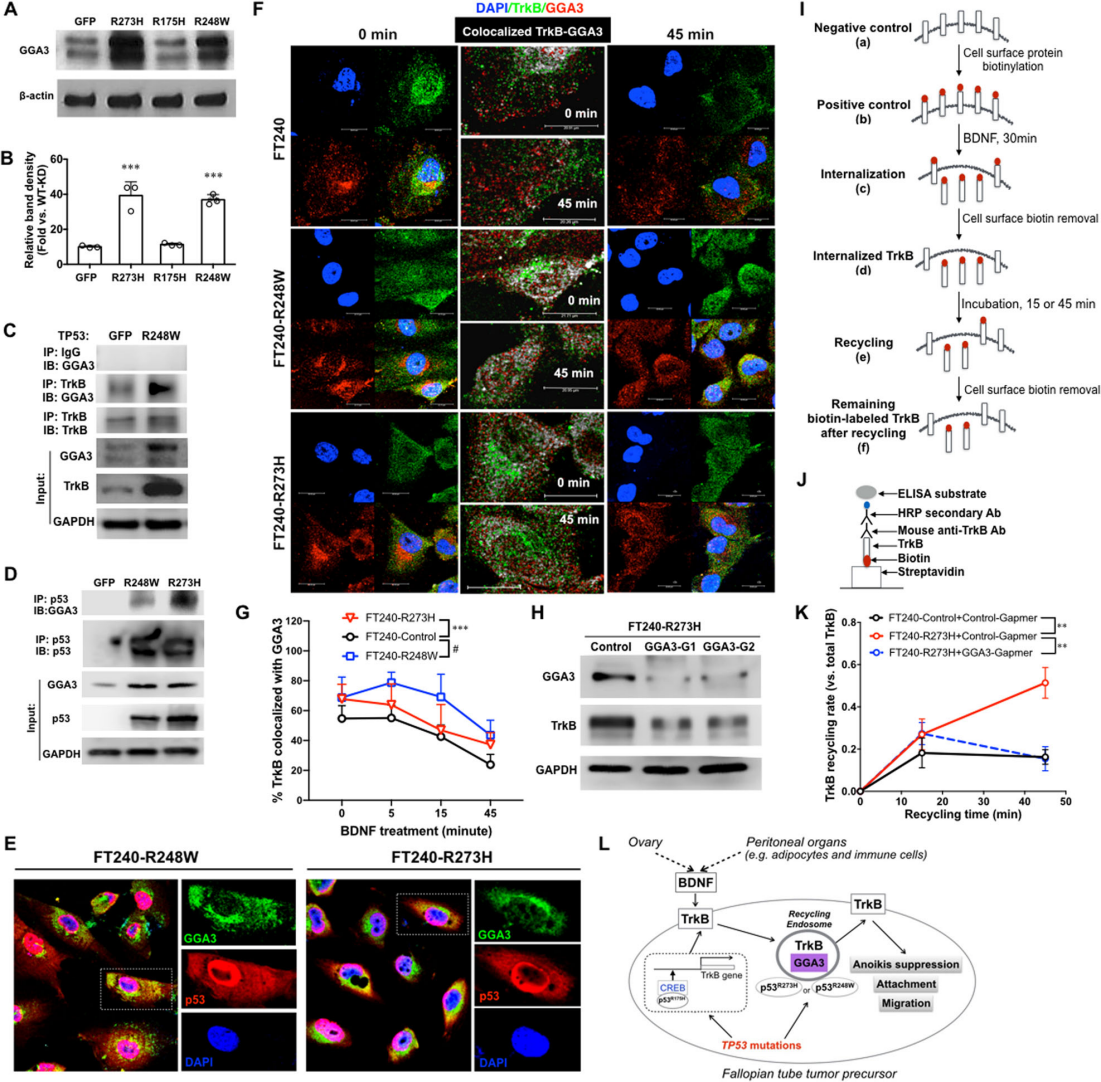
Mutant p53 promotes GGA3-regulated TrkB recycling in FTE cells. **a** Representative images of GGA3 western blot show upregulation of GGA3 in FT240 cells expressing p53-R273H and R248W, but not p53-R175H. The same membrane as in Fig. 1a was re-blotted for GGA3 and β-actin loading control. **b** The density of GGA3 bands of FTE240 cells expressing GFP or mutant p53 was quantified. *n* = 3. ***p < 0.0005 for one-way ANOVA followed by Tukey’s HSD test. **c** Coimmunoprecipitation (co-IP) of TrkB and GGA3 in FT240 cells expressing GFP or mutant p53-R248W. **d** Co-IP of GGA3 and mutant p53-R248W or R273H in FT240 cells expressing GFP, mutant p53-R248W, or p53-R273H. **e** IF staining of mutant p53 (red) and GGA3 (green) in FT240 cells expressing mutant p53-R248W or p53-R273H. DAPI (blue) was used to stain nuclear DNA. **f** IF staining of TrkB (green) and GGA3 (red). DAPI was used to stain nuclear DNA. The colocalized TrkB and GGA3 signals were labeled as white in the middle panel. Cells were treated with 50 ng/ml BDNF for 0–45 min. **g** TrkB-GGA3 colocalization was quantified by analyzing about 20 cells in 5–6 images at each time point using ImageJ software. Mutant p53 is associated with higher levels of TrkB-GGA3 colocalization in FTE cells. ***p < 0.0005 and ^#^*p* < 0.0001 for two-way ANOVA followed by Sidak HSD test. **h** Western blot images validated the knockdown of GGA3 by two GapmeRs (GGA3-G1 and G2). GGA3-G1 and G2 decreased the level of TrkB protein compared to the negative control GapmeR. **i** Schematic flow chart of TrkB recycling assay using biotin-labeling. The red dots indicate biotin. Surface proteins of FTE cells were biotinylated with membrane impermeable sulfo-NHS-LC-LC-Biotin (a, b). BDNF treatment for 30 min induced the internalization of biotin-labeled TrkB (b, c). Cell surface biotin was removed with biotin-removal buffer on ice (c, d) followed by a rewarming step at 37 °C to allow TrkB to recycle to cell surface (d, e). A second round of biotin-removal was performed (e, f). **j** Schematic diagram of biotin-labeled TrkB ELISA (enzyme-linked immunosorbent assay). The biotin-labeled TrkB protein was captured by a streptavidin-coated 96-well plate and quantified with TrkB ELISA. **k** TrkB recycling rate was higher in FT240 cells expressing p53-R273H than the control FT240 cells. Knockdown of GGA3 by the mix of two Gapmers suppressed the recycling of TrkB. *n* = 3. **p < 0.005 for two-way ANOVA followed by Sidak HSD test. **l** Schematic model of BDNF/TrkB activation in fallopian tube tumor precursors that express mutant p53.

In support of this hypothesis, GGA3 protein level was significantly higher in FTEs that overexpressed p53R248W or p53R273H compared to the control FTEs that overexpressed GFP (Fig. 4a, b). p53R248W enhanced the direct interaction of TrkB and GGA3 (Fig. 4c). Co-IP and confocal microscopy data demonstrated that p53R248W and p53R273H directly interacted and colocalized with GGA3 (Fig. 4d, e). These results led us to test the hypothesis that p53R248W and p53R273H modulate the GGA3-regulated TrkB receptor recycling.

### p53R248W and p53R273H enhance GGA3-regulated TrkB recycling

Using TrkB-GGA3 double staining and quantification of their colocalization (Fig. 4f and Supplementary Fig. S15), we assessed TrkB-GGA3 interaction during endocytic recycling. In FT240 cells, the portion of TrkB that colocalized with GGA3 started to decrease after 5 min of BDNF treatment, while the p53R248W or p53R273H-expressing FT240 cells sustained a higher level of GGA3-TrkB colocalization than the control FT240 cells, indicating enhanced endocytic recycling of TrkB (Fig. 4g).

Next, we knocked down the expression of GGA3 using locked nucleic acid-conjugated chimeric single-strand antisense oligonucleotides that are similar to siRNAs for silencing gene expression, also called “GapmeRs”^63^, which also downregulated TrkB protein in the p53R273H-expressing FTEs (Fig. 4h) and demonstrated the critical role of GGA3 in maintaining TrkB stability.

Finally, we assessed the recycling rate of TrkB using a method illustrated in Fig. 4i, j. The recycling of TrkB was stimulated upon BDNF treatment. TrkB recycling rate in the p53R273H-expressing FT240 cells was higher than the control FT240 cells. GGA3 GapmeRs decreased the recycling rate of TrkB in the p53R273H-expressing FTEs to the level similar to that of the control FT240 cells (Fig. 4k).

Once more, these data support the hypothesis that mutant p53, such as R248W and R273H, enhances the recycling and stability of TrkB. This mechanism is different from the transcriptional activation of TrkB by p53R175H; however, it still leads to the enhanced oncogenic functions of BDNF/TrkB pathway (illustrated as a schematic model in Fig. 4l).

### Enhanced BDNF/TrkB signaling promotes FTE tumor precursors survival and attachment in vivo

**Fig. 5:**
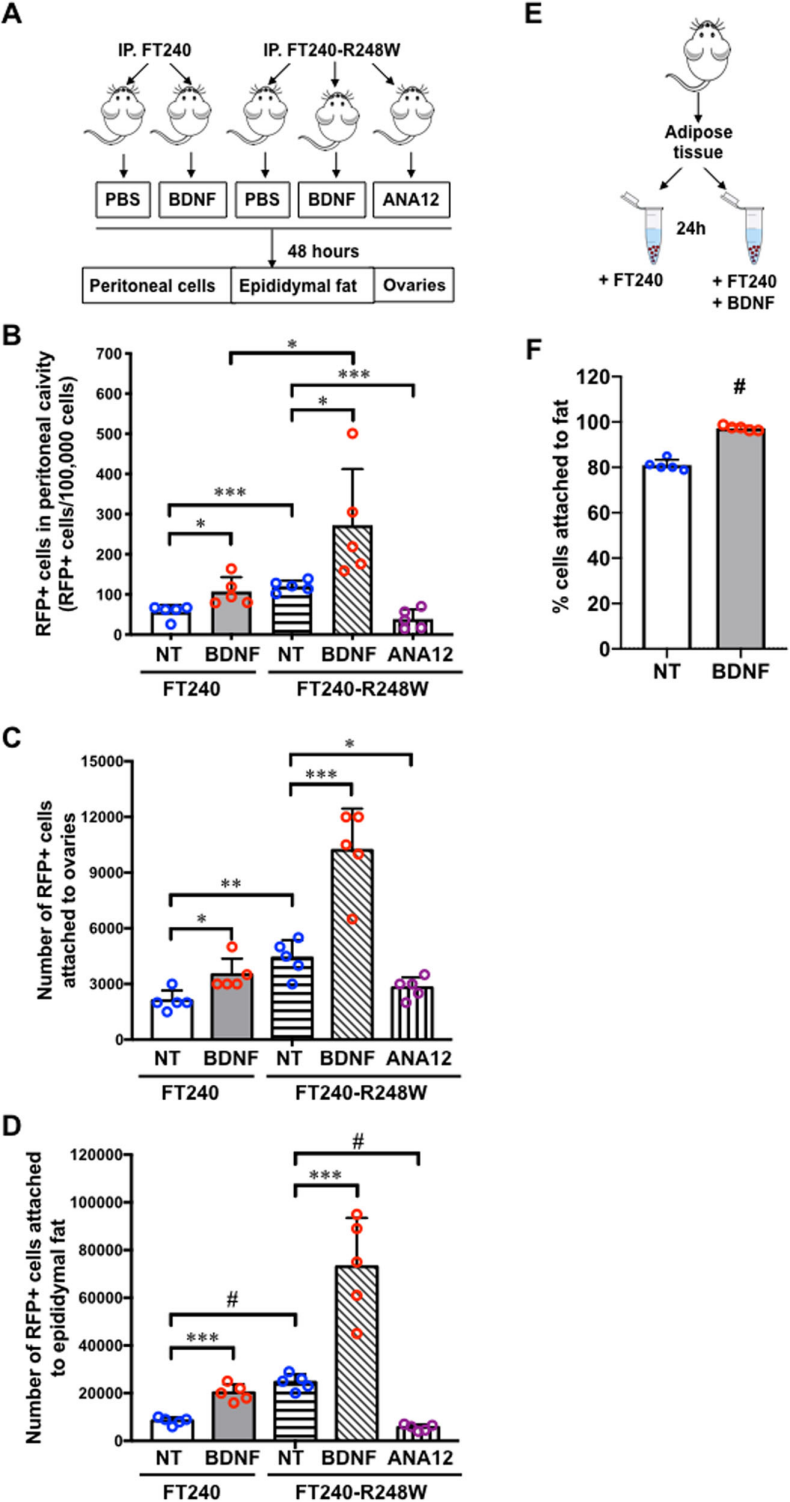
BDNF enhances the survival of FTE cells in vivo. **a** Schematic flow chart of mice injected with red fluorescent protein positive (RFP+) FT240 cells or p53-R248W-expressing FT240. FT240 cells, BDNF (0.2 ng/mouse), ANA-12 (0.5 mg/kg), and/or phosphate-buffered saline (PBS) were injected intraperitoneally to nude mice. *n* = 5. RFP+ cells in the peritoneal cavity were assessed by flow cytometry. RFP+ cells attached to the ovaries or epididymal fat pads were assessed by manual cell counting of the dissociated tissues. **b**−**d** The numbers of RFP+ FT240 cells in the peritoneal cavity (**b**), attached to the ovaries (**c**), and attached to the epididymal fat pads (**d**) were increased by BDNF treatment and deceased by ANA-12 treatment. *p < 0.05, **p < 0.005, ***p < 0.0005, and ^#^*p* < 0.0001 for unpaired Student’s *t* test. **e** Schematic flow chart illustrating the co-culture of adipose tissues and FT240 cells. **f** BDNF (50 ng/ml) increased the percentage of FT240 cells attached to the adipose tissues. *n* = 5. ^#^*p* < 0.0005 for unpaired Student’s *t* test.

We injected RFP-labeled (RFP+) FT240 cells intraperitoneally to nude mice (Fig. 5a) and detected 56 ± 8 RFP+ cells in every 10^5^ peritoneal cells of the control mice after 48 h by flow cytometry. When we also injected 200 pg BDNF in 100 μl phosphate-buffered saline (PBS) to each mouse to reach about 1 ng/ml in the peritoneal fluid, we detected 107 ± 16 RFP+ cells per 10^5^ peritoneal cells, which was significantly more than the control group (Fig. 5b and Supplementary Fig. S16). BDNF treatment also increased the numbers of RFP+ cells attached to the ovaries and epididymal adipose tissues (Fig. 5c, d).

When we injected the same number of RFP+ p53R248W-expressing FT240 cells, we detected 121 ± 6 RFP+ cells per 10^5^ peritoneal cells, which was higher compared to the control FT240 cells. Significantly higher numbers of cells survived in the BDNF-treated p53R248W-expressing FT240 group than the control group (Fig. 5b). ANA12 (0.5 mg/kg) injection significantly decreased the number of peritoneal p53R248W-expressing FT240 cells to 39 ± 11 cells per 10^5^ cells (Fig. 5b). A similar pattern was demonstrated in RFP+ cells attached to the ovaries and adipose tissues (Fig. 5c, d). Therefore, p53R248W contributed to enhancing the survival of FTEs when they disseminated and traveled throughout the peritoneal cavity. TrkB inhibition by ANA12 suppressed the survival of FTEs. The activity of BDNF to promote FTEs attachment was also demonstrated in a co-culture model of FTEs and adipose tissues (Fig. 5e, f).

### BDNF induces prosurvival and prometastatic gene expression to promote tumor formation

**Fig. 6:**
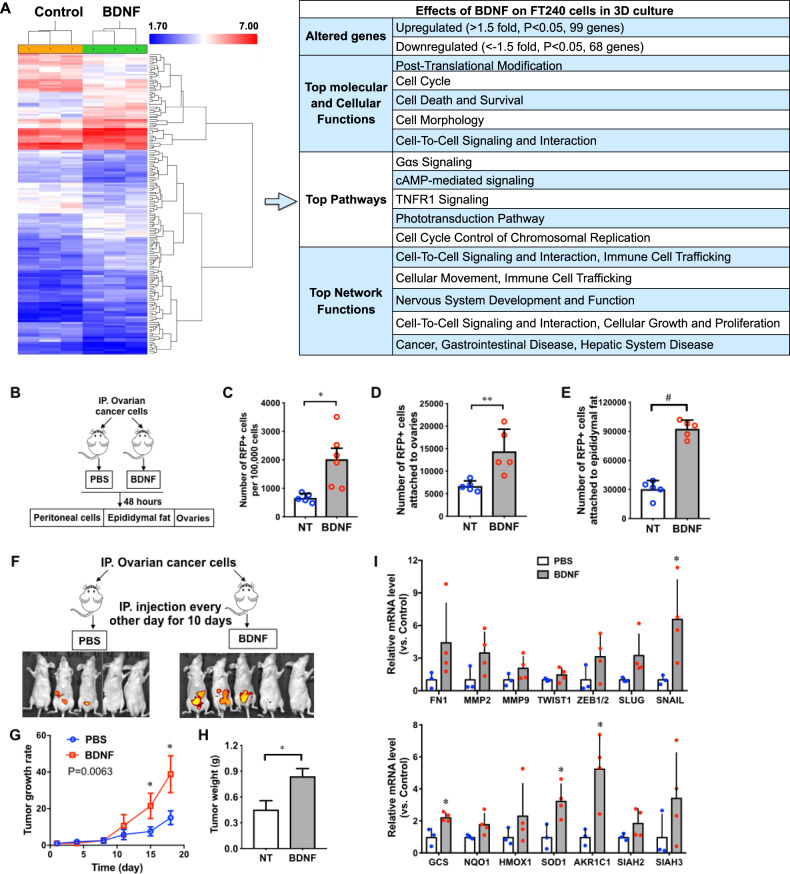
BDNF promotes the expression of prosurvival and prometastasis genes in vitro and in vivo. **a** Heat map and Ingenuity Pathway Analysis (IPA) of human transcriptome array of untreated (NT) and BDNF-treated FT240 cells in 3D culture for 24 h. *n* = 3. *Network functions score was determined by number of molecules, cellular, and disease processes predicted to be affected by differentially regulated genes in BDNF-treated FT240 cells vs. control cells as determined by the IPA software. **b** Schematic flow chart of mice injected with RFP+ ovarian cancer (OVC) cells. **c**–**e** BDNF (0.2 ng/mouse) treatment increased the numbers of RFP+ OVC cells in the peritoneal cavity (**c**), attached to the ovaries (**d**), and attached to the fat tissues (**e**). *n* = 5. *p < 0.05, **p < 0.005, and ^#^*p* < 0.0005 for unpaired Student’s *t* test. **f** Schematic flow chart of mice injected with RFP+ ovarian cancer (OVC) cells. OVC cells and BDNF (0.2 ng/kg) were injected intraperitoneally to nude mice. *n* = 5. Representative images of in vivo live imaging of mice 10 days after injection. **g** Tumor growth curves of mice injected with RFP+ OVC cells. Two-way ANOVA followed by Sidak’s test. **h** Weight of tumors from mice injected with RFP+ OVC cells. Student’s *t* test, *p < 0.05. **i** RT-QPCR data of tumors from mice injected with PBS (untreated, NT, *n* = 3) or BDNF (*n* = 4). *p* < 0.0001 between the PBS and BDNF groups, two-way ANOVA followed by Sidak HSD test, *p < 0.05 between the PBS and BDNF groups for each gene.

Using Human Transcriptome Array, we identified that 24-h BDNF treatment of FTE240 cells cultured in 3D condition altered the expression of 167 genes with known functions over 1.5 folds compared to the untreated control cells, of which 99 genes were upregulated and 68 genes were downregulated (Fig. 6a and Supplementary Table). Ingenuity Pathway Analysis (IPA) of these genes suggests that they were involved in post-translational modification, cell cycle, cell death and survival, cell morphology, and cell-to-cell signaling and interaction. The top pathways and network functions affected by these genes also included cellular movement and proliferation. BDNF treatment also induced the expression of prosurvival and prometastatic genes, such as epithelial-to-mesenchymal transition (EMT) genes, FN1 (fibronectin-1), MMP2, MMP9, TWIST1, ZEB1/2, SLUG, and SNAIL (Supplementary Table). The involvement of EMT allows cancer cells to overcome anoikis and travel to metastatic sites^64,65^. NRF2-regulated antioxidant response genes^66^, including GCS, NQO1, HMOX1, SOD1 and AKR1C1, were also induced by BDNF treatment (Supplementary Table) Our data suggest the role of BDNF as an activator of antioxidant response pathway^67,68^. Seven-in-absentia homolog (SIAH) protein family members, SIAH1, SIAH2 and SIAH3, were upregulated by BDNF. They have been implicated in regulating cellular response to hypoxia^69^.

We confirmed some of these findings in a xenograft mouse model using a patient-derived ovarian cancer (OVC) cell line with ectopic overexpression of mutant p53R175H. We demonstrated that BDNF promoted tumor formation and induced prosurvival and prometastatic gene expression in vivo. When RFP+ OVC cells were injected into nude mice (Fig. 6b), BDNF treatment increased the numbers of RFP+ OVC cells in the peritoneal cavity, the ovaries, and the epididymal fat after 48 h (Fig. 6c, d, e and Supplementary Fig. S17). When the injected OVC cells we allowed to form tumors, the BDNF-treated mice (200 pg/mouse, every other day) developed significantly larger tumors than the control mice (Fig. 6f, g, h).

The RNA expression profiles of these tumors confirmed the BDNF-induced upregulation of EMT genes, NRF2 target genes and SIAH genes that were identified in FTEs in vitro (Fig. 6i). Our data suggested their roles as downstream genes of BDNF.

## Discussion

Our data suggest that BDNF in the ovarian FF protects FTE tumor precursors and may contribute to the progression from FT lesions into widespread high-grade serous carcinomas. We also identified a previously unrecognized GOF of mutant p53 in enhancing BDNF/TrkB signaling.

HGSOC is characterized by a high frequency of p53 mutation, chromosomal instability, and DNA copy number aberrations^20–22^. Molecular profiles uncover that HGSOCs are more similar to STIC lesions than ovarian surface epithelium or ovarian cysts in terms of their immmunohistochemical, transcriptomic, and genetic phenotypes^10,11,13–15,70^. The presence of *TP53* mutations in the FTE tumor precursors^13,15,70^ indicates that the acquisition of *TP53* mutation occurs as an early tumorigenic event critical for HGSOC formation. However, p53 mutation alone is not sufficient to transform FTEs and cause HGSOC^71^. We hypothesize that the interplays of p53 mutation with other intrinsic and extrinsic factors during the pathological processes are crucial for driving HGSOC initiation and progression. We have provided evidence that GOF p53 mutations promote TrkB transcription or recycling in FTE tumor precursors leading to their enhanced responses to BDNF in the distal tubal microenvironment. It demonstrated that growth factors or stress signals from the ovary could synergize with these *TP53* mutations to facilitate the development of tumor precursors towards invasive HGSOC.

Our study focuses on the comparison between LOF p53 mutation and three most common GOF p53 mutations and supports the hypothesis that these *TP53* GOF mutations subvert BDNF/TrkB signaling to promote the development of HGSOC. It is noted that mutant p53 can induce similar phenotypic changes via distinctive mechanisms, such as increasing transcriptional activity of CREB or receptor recycling by GGA3 both leading to enhanced TrkB. The impacts and mechanisms of other p53 mutations on BDNF/TrkB pathway are yet to be studied. It has been shown that p53R175H and p53R273H enhance MET receptor recycling to promote cancer cell invasion^72^. Since the recycling of MET is also regulated by GGA3^61^, our findings of mutant p53-GGA3 interaction may also explain the mutant p53-enhanced MET recycling.

Our recent study of primary and recurrent HGSOC tumor specimens showed that the mutation features in matched primary-metastatic tumors were extremely similar and the synchronous bilateral ovarian cancers shared the majority of their somatic mutations indicating a common origin^73^. These findings suggest that FTE tumor precursors gained the metastatic ability at early stages of tumor development. The detachment of FTE tumor precursors and their survival in an anchor-independent condition are critical steps that lead to tumor spreading to the ovary and peritoneal organs. Enhanced activation of TrkB can prolong the survival and active migration of FTE during dissemination, thereby increasing probability to attach to other organs.

BDNF in the ovary is critical for the development of oocyte into preimplantation embryos^41,42^. High levels of BDNF are correlated with endometriosis and pelvic pain^74^. We identified the expression of TrkB in the FTEs, which had not been reported previously. To date, the physiological functions of TrkB in the FTEs have not been investigated. Currently, we are generating an oviduct epithelium-specific TrkB-knockout mouse model for understanding the role of FTE TrkB in the development and physiology of female reproductive system.

BDNF and TrkB are upregulated in cancers and promote tumor progression^45–53^. Gene fusion abnormalities of TrkB are also common in multiple cancer types, resulting in the production of chimeric proteins with constitutively activated or overexpressed oncogenic kinase function^75^. Our findings add that BDNF/TrkB signaling can contribute to the early initiation of HGSOC from FTE tumor precursors, in particular, when enhanced by certain TP53 mutations. It is important to note that BDNF is also expressed by omental adipocytes, peritoneal visceral epithelial cells, and multiple types of immune cells in the omentum and peritoneum^76–78^, where HGSOCs commonly spread. Therefore, BDNF/TrkB signaling may facilitate the peritoneal metastasis of HGSOC.

BDNF/TrkB pathway dysregulation has been implicated in the pathogenesis of neurodegenerative diseases, psychiatric disorders, and metabolic syndromes^40^. The therapeutic use of BDNF and its mimics are under development^79–81^. BDNF overexpression in the hypothalamus can also increase antitumor immune response^82,83^. The coexistence of neuroprotective and oncogenic effects of BDNF raises a number of pharmacokinetic and safety issues for the clinical uses of BDNF/TrkB pathway modulators. It is clear that localized delivery of these drugs to the target organs will be critical for achieving maximal efficacy, such as activating TrkB in neurons with BDNF mimics or reducing tumor burden and preventing metastasis with TrkB antagonists.

The molecular mechanisms revealed in this study provided insights into the interactions between GOF mutant p53 and the tubal/ovarian microenvironment through BDNF/TrkB oncogenic pathway that can facilitate tumor spreading in all stages of HGSOC development and progression. Therefore, a better understanding of these pathways will contribute to the discovery of new biomarkers and therapeutic targets.

## Materials and methods

### Reagents and cell lines

FTE cell lines were generated as previously described^56^. FTE cell lines expressing shRNA-resistant mutation p53 were generated by lentivirus transduction as described in Supplementary Methods. Granulosa cell line KGN was provided by Dr. Yingqun Huang (Yale University). The ovarian cancer cell line (OVC) was derived from a serous epithelial ovarian cancer sample and provided by Dr. Gil Mor (Yale University). This cell line stably overexpresses mutant p53R175H via a lentiviral vector. FTE cell lines, FT240, FT246, and FT340, were propagated in DMEM/F12 medium. KGN and OVC cells were cultured in RPMI 1640 medium. Detailed procedures for cell culture and conditional medium collection are described in Supplementary Methods. Chemicals, antibodies, and primers were described in the supplementary information.

### Western blot, flow cytometry and immunofluorescence (IF) staining

Details of western blot, flow cytometry and IF staining and the information of antibodies are listed in the supplementary information.

### Caspase-3 activity, cell viability and anoikis-recovery assays

Caspase-3 activity, cell viability and anoikis-recovery assays were performed using Caspase-Glo 3/7 Assay, CellTiter-Glo 2D or 3D Cell Viability Assay (Promega, Madison, WI) as described in detail in the Supplementary Methods. In the anoikis-recovery assay, cells were cultured in ultra-low attachment plates for 24 h before they were re-plated to the collagen I-coated 96-well plates to recover for 48 h.

### Hydrogel migration assay

Migration assay was performed using Cell-Mate 3D Gel 40 Kit (BRTI Life Sciences, Two Harbors, MN) as described in detail in the Supplementary Methods. One million cells were added to each piece of gel and cultured in medium with BDNF (50 ng/ml) or conditional medium (50%). The migrated cells outside the gel were stained by crystal violet or quantified with cell viability assay.

### 3D cell adhesion assay

SphereCol human type I collagen-coated beads (Advanced BioMatrix, San Diego, CA) were incubated with FTEs (10^5^ cells/13.9 mg beads/ml) on a rotator in 37 °C incubator for desired time. Cells that did not attach to the beads were washed off with PBS after incubation. The attached cells were quantified using CellTiter-Glo assay. Details are described in the Supplementary Methods.

### Gene knockdown with shRNAs and GapmeRs

TrkB-shRNA lentiviral particles (#TL320436V, OriGene Technologies, Rockville, MD) were transduced following the manufacturer’s instruction. TrkB knockdown was validated by western blot. GapmeRs targeting GGA3 and the negative control GapmeR (#LG00227196-DDA, LG00227197-DDA, and LG00000002-DDA, Qiagen, Germantown, MD) were delivered to cells via gymnosis following the manufacturer’s instruction.

### Follicular fluid (FF) collection and in vitro tests

FF collection protocol was approved by Yale University Human Research Protection Program Institutional Review Boards. De-identified FF samples were collected during oocyte retrieval of women undergoing in vitro fertilization by standard ovarian stimulation protocols. Each FF sample was collected from one woman. The FF was filtered through a 0.45 μm filter and diluted with Opti-MEM medium (1:1) for in vitro assays.

### Quantitative real-time PCR (QPCR)

Total RNA was extracted using RNA Purification Kit (Norgen Biotek, Thorold, ON, Canada). cDNA was synthesized with qScript SuperMix (Quantabio, Beverly, MA). QPCR was performed using SYBR Green Supermix and CFX Connect detection system (Bio-Rad, Hercules, CA). GAPDH was used as a reference gene in the ΔΔCT expression analysis. All reactions were performed with at least three biological replicates.

### Co-immunoprecipitation (co-IP) and chromatin immunoprecipitation (CHIP)

In co-IP and CHIP assays, SureBeads (Bio-Rad) were incubated with 1 µg antibody for 10 min at room temperature to prepare the antibody-conjugated beads. Detailed co-IP and CHIP-QPCR protocols are described in the Supplementary Methods.

### TrkB recycling assay

FTE cells were starved for 2 h, before they were labeled by sulfo-NHS-S-S-biotin (ProteoChem, Hurricane, UT). The unreacted biotin was quenched with Tris-buffered saline. Cells were incubated with 50 ng/ml BDNF at 37 °C for 30 min to induce TrkB internalization. The remaining cell-surface biotin was removed in biotin-removal buffer for 15 min at 4 °C. Cells were then rewarmed and incubated at 37 °C to allow receptor recycling. A second round of biotin removal was performed before the cells were lysed with ELISA lysis buffer (Cell Signaling Technology, #9803). The biotin-labeled TrkB was quantified by ELISA using streptavidin-coated 96-well plates (Eagle Biosciences, Amherst, NH), anti-TrkB antibody, HRP-conjugated secondary antibody, and SuperBrite HRP Chemiluminescence Substrate (BioVision, Milpitas, CA). TrkB recycling rate is calculated by subtracting the remaining biotin-labeled TrkB after the final biotin removal from the sample after recycling, and then normalizing to the amount of total internalized TrkB before recycling. Details of recycling assay are described in the Supplementary Methods.

### Human transcriptome array and IPA analysis

FTE cells were treated with BDNF 50 ng/ml in 3D culture for 24 h. Untreated cells were cultured under the same condition as control. RNA was analyzed using Clariom D Human Transcriptome Array and Transcriptome Analysis Console (Thermo Fisher Scientific, Waltham, MA). Details of the analysis are described in the Supplementary Methods.

### Mouse models

Animal experiments were approved by Yale University Institutional Animal Care and Use Committee. Details are described in the Supplementary Methods. Briefly, red fluorescent protein (RFP)-labeled cells were intraperitoneally injected to athymic nude mice (3 × 10^6^ cells/mouse). Then BDNF (0.2 ng/mouse) or ANA-12 (0.5 mg/kg) was injected intraperitoneally. Forty-eight hours after injection, cells were collected from the peritoneal lavage fluid as previously described^84^. RFP+ cells were detected using flow cytometry. In the tumor formation model, 10^6^ OVC cells were intraperitoneally injected to nude mice. BDNF (0.2 ng/mouse) was intraperitoneally injected every other day for 10 days. Tumor growth was monitored by Spectrum In Vivo Imaging System (PerkinElmer, Waltham, MA) twice a week. In ex vivo adhesion assay, 10 mg epididymal adipose tissues were co-cultured with 100,000 RFP-labeled FTE cells in the presence or absence of 50 ng/ml BDNF. The numbers of cells that attached to the adipose tissues were determined by subtracting the numbers of cells left in the suspension from the total cell numbers.

### Statistics

Numerical values are presented as mean ± SD. For comparisons between two groups, *p* values were calculated using paired or unpaired two-tailed Student’s *t* tests. One-way ANOVA was used to analyze more than two independent groups. Two-way ANOVA was used to compare the difference in experiments with two independent variables. Details of data analysis are described in the Supplementary Methods.

## Supplementary information

Supplemental Methods

List of primer, antibodies, and drugs

Supplementary figures

Supplemental Table
