# Supplementary material for: Ovarian BDNF promotes survival, migration, and attachment of tumor precursors originated from p53 mutant fallopian tube epithelial cells
Source: Oncogenesis. 2020 May 29;9(5):55. doi: 10.1038/s41389-020-0243-y (PMC7260207; doi:10.1038/s41389-020-0243-y)
Supplement: Supplementary file 1 — Supplemental Methods [file 41389_2020_243_MOESM1_ESM.docx]

***Supplemental Methods:***

*Summary: This section includes the details of experimental procedures and materials that are not described in the Method Section.*

**Construction of shRNA resistant mutant p53 plasmids**

For constructing the shRNA resistant mutant p53 lentvirus plasmids, we obtained the mutant p53 plasmids from Addgene (Watertown, MA). Plasmid constructs of mutant p53R175H and R273H were gifts from Bernard Futscher (Addgene plasmid # 22936 ; http://n2t.net/addgene:22936 ; RRID:Addgene_22936 and Addgene plasmid # 22934 ; http://n2t.net/addgene:22934 ; RRID:Addgene_22934). pCMV-Neo-Bam p53 Plasmid constructs of mutant p53R248W was a gift from Bert Vogelstein (Addgene plasmid # 16437 ; http://n2t.net/addgene:16437 ; RRID:Addgene_16437). p53R248W was cloned into the same vector as p53R175H and R273H. pLenti6-GFP was a gift from Daniel Haber (Addgene plasmid # 35637 ; http://n2t.net/addgene:35637 ; RRID:Addgene_35637).

The p53-shRNA in the FTE cell lines was pMKO.1 puro p53 shRNA 1 (Addgene plasmid # 10671 ; http://n2t.net/addgene:10671 ; RRID:Addgene_10671) as previously described protocols for immortalizing human FTEs (54). This shRNA sequence is “ccggt**gactccagtggtaatctac**ttcaagagagtagattacactggagtctttttg”. The underlined sequence targets TP53 transcript (776-794). We modified this sequence into “gatagttccgggaacttgc” by PCR resulting their resistance to the p53 shRNA without changing the amino acid products due to the alternative codons. (Sequences shown below)

[501-900] Wild-type p53 (WT): gcacatgacggaggttgtgaggcgctgcccccaccatgagcgctg

shRNA resistant p53-R175H (R175H): gcacatgacggaggttgtgaggCACtgcccccaccatgagcgctg

shRNA resistant p53-R248W (R248W): gcacatgacggaggttgtgaggcgctgcccccaccatgagcgctg

shRNA resistant p53-R273H (R273H): gcacatgacggaggttgtgaggcgctgcccccaccatgagcgctg

(WT): ctcagatagcgatggtctggcccctcctcagcatcttatccgagtggaaggaaatttgcgtgtggagtatt

(R175H): ctcagatagcgatggtctggcccctcctcagcatcttatccgagtggaaggaaatttgcgtgtggagtatt

(R248W): ctcagatagcgatggtctggcccctcctcagcatcttatccgagtggaaggaaatttgcgtgtggagtatt

(R273H): ctcagatagcgatggtctggcccctcctcagcatcttatccgagtggaaggaaatttgcgtgtggagtatt

(WT): tggatgacagaaacacttttcgacatagtgtggtggtgccctatgagccgcctgaggttggctctgactgt

(R175H): tggatgacagaaacacttttcgacatagtgtggtggtgccctatgagccgcctgaggttggctctgactgt

(R248W): tggatgacagaaacacttttcgacatagtgtggtggtgccctatgagccgcctgaggttggctctgactgt

(R273H): tggatgacagaaacacttttcgacatagtgtggtggtgccctatgagccgcctgaggttggctctgactgt

(WT): accaccatccactacaactacatgtgtaacagttcctgcatgggcggcatgaaccggaggcccatcctcac

(R175H): accaccatccactacaactacatgtgtaacagttcctgcatgggcggcatgaaccggaggcccatcctcac

(R248W): accaccatccactacaactacatgtgtaacagttcctgcatgggcggcatgaacTGGaggcccatcctcac

(R273H): accaccatccactacaactacatgtgtaacagttcctgcatgggcggcatgaaccggaggcccatcctcac

(WT): catcatcacactggaa**gactccagtggtaatctac**tgggacggaacagctttgaggtgCGTgtttgtgcct

(R175H): catcatcacactggaaGATAGTTCCGGGAACTTGctgggacggaacagctttgaggtgcgtgtttgtgcct

(R248W): catcatcacactggaaGATAGTTCCGGGAACTTGctgggacggaacagctttgaggtgcgtgtttgtgcct

(R273H): catcatcacactggaaGATAGTTCCGGGAACTTGctgggacggaacagctttgaggtgCACgtttgtgcct

(Modified shRNA resistant region)

(WT): gtcctgggagagaccggcgcacagaggaagagaatctccgcaagaaaggggagcctcaccacgagctgccc

(R175H): gtcctgggagagaccggcgcacagaggaagagaatctccgcaagaaaggggagcctcaccacgagctgccc

(R248W): gtcctgggagagaccggcgcacagaggaagagaatctccgcaagaaaggggagcctcaccacgagctgccc

(R273H): gtcctgggagagaccggcgcacagaggaagagaatctccgcaagaaaggggagcctcaccacgagctgccc

*Note: Codons highlighted in red are the hotspot mutations.

The PCR products with modified sequences were cloned into the backbone of lentivirus vector of pLenti6/V5-p53_wt p53 that was a gift from Bernard Futscher (Addgene plasmid # 22945 ; http://n2t.net/addgene:22945 ; RRID:Addgene_22945).

**Lentivirus transduction for mutant p53 overexpression**

Mutant p53 lentiviruses were produced by transfecting HEK 293T cells with polyethylenimine (PEI). HEK 293T cells were seeded in a 10 cm plate at a density of 5 × 10^6^ cells. When confluence reached 70%, transfection was performed by adding a mixture of 10 μg plasmid DNA (5:2:3, psPAX: pMD2G: plenti-mutant p53 or plenty-GFP) and 30 μg PEI. Virus was harvested after 48 h and concentrated by ultracentrifugation. Viral infection was done in suspension using 10^6^ FTE cells in 1 mL of fresh RPMI 1640 media with concentrated viral particles. Cells were incubated 1 h at 37°C, shaking intermittently. After incubation the cells were transferred to a T25 tissue culture flask by adding 4 mL of fresh media. The next day, the viral media was removed and replaced with fresh RPMI to allow the cells to recover. Forty-eight hours after adding the virus, Blasticidin was added to the medium to select the positive cells. Western blot was performed with the FTEs to confirm the overexpression of mutant p53.

**Cell culture and conditional medium**

FTE cell lines were propagated in DMEM/F12 medium. Epithelial ovarian cancer cell line and KGN granulosa cell line were cultured in RPMI 1640 medium. Both media contain 10% fetal bovine serum (FBS), L-glutamine, and penicillin/streptomycin. Cells were cultured in an incubator at 37°C with 5% CO_2_ and 95% humidity. To collect the KGN conditional medium, they were cultured in Opti-MEM reduced serum medium (Life Technologies, Carlsbad, CA, USA) for 48 hours. The 3D-cultured cells were seeded in 96-well ultra-low attachment plates (Greiner Bio-One, Monroe, NC, USA) at a density of 3000 cells/well in Opti-MEM reduced serum medium. Cell lines were authenticated by STR DNA profiling and tested for mycoplasma contamination regularly.

**Western blot**

Cell lysates prepared with lysis buffer (1% Triton X-100, 0.05% SDS, 100 mM Na_2_HPO_4_, and 150 mM NaCl) were electrophoresed in a 12% SDS-polyacrylamide gel and transferred onto 0.45 μm PVDF membranes (GE Healthcare, Chicago, IL, USA). After incubation in blocking buffer (5% BSA, 1X TBS, 0.1% Tween-20), the membranes were incubated with primary antibodies at 4ºC overnight followed by the HRP-conjugated secondary antibodies for 1 hour at room temperature. Blots were developed using ECL Blotting Substrates (Bio-Rad, Hercules, CA, USA) and imaged using an Amersham 600 imager (GE Healthcare, Chicago, IL, USA).

**Flow cytometry**

Cells were trypsinized and resuspended in PBS. After cells were fixed in 4% formaldehyde for 15 minutes at room temperature, they were permeabilized in ice-cold 100% methanol and washed with excess PBS. One million cells were aliquoted for staining with 100 µl of diluted primary antibody in incubation buffer for 1 hour at room temperature. After washing twice with incubation buffer, cells were resuspended in 100 µl of diluted fluorochrome-conjugated secondary antibody to incubate for 30 min at room temperature and washed twice with PBS before analysed on the flow cytometer.

**Immunofluorescence (IF) staining**

Formalin-fixed paraffin-embedded (FFPE) tissue sections were deparaffinized and hydrated. Slides were submersed in 1X citrate unmasking solution and heated in a microwave to incubate at 95-98°C for 10 min. After cooling, the slides were incubated in blocking buffer (5% normal serum and 0.3% Triton X-100 in 1X PBS) for 60 min at room temperature. For staining cultured cells, the cells were cultured on chamber slides, fixed with 4% paraformaldehyde in PBS for 10 min at room temperature, and then washed with PBS three times followed by incubation in blocking buffer. Primary antibodies were diluted in antibody dilution buffer (1% BSA and 0.3% Triton X-100 in 1X PBS) and incubated with the slides overnight at 4°C. After rinsing with PBS fluorochrome-conjugated secondary antibody was added and incubated for 1 hour at room temperature in the dark. Prolong Gold Antifade Reagent with DAPI was used to mount the slides (#9071, Cell Signaling, Danvers, MA, USA). The stained slides were imaged using Leica SP8 Laser Scanning Confocal microcope. The images were analysed using the colocalization function of ImageJ Software (2.0.0).

**Cell viability assay**

Cell viability was assessed using CellTiter-Glo 2D or 3D Cell Viability Assay (Promega, Madison, WI) according to the manufactures’ instruction. Briefly, CellTiter-Glo Reagent equal to the volume of cell culture medium was added to the cells. The plate was shaken for 5-10 minutes and incubated at room temperature for 25 minutes. The luminescence signal was recorded using a GloMax Navigator Microplate Luminometer (Promega).

**Anoikis-recovery cell viability assay**

Cells were trypsinized from cell culture dishes and plated in ultra-low attachment plates to induce anoikis. After 24 hours, cells were re-plated (as 3000 cells/well) in collagen I-coated 96-well plates to recover for 48 hours. Cell viability of the recovered cells was determined using CellTiter-Glo assay. The collagen I-coated plate was prepared by incubating plates with 500ug/ml collagen I solution (in 0.02M acetic acid) for 2 hours at 37ºC and rinsing with PBS.

**Caspase-3 activity assay**

Caspase-3 activity was evaluated using Caspase-Glo 3/7 Assay kit (Promega) according to the manufacturer’s instruction. Briefly, 10 μg protein lysate was diluted to a final volume of 50 μL. An equal volume of Caspase-Glo® 3/7 Reagent was added to the lysate and incubated at room temperature for 1 hour. The luminescence signal in each well was recorded using a Navigator Microplate Luminometer.

**Hydrogel migration assay**

Migration assay was performed suing Cell-Mate 3D Gel 40 Kit (BRTI Life Sciences, Two Harbors, MN). One million cells were resuspended in 40 μl hydration fluid and added to the dry 3D gel. After incubating in room temperature for 5 minutes, the gel piece was transferred to a well in a 6-well plate with Opti-MEM medium. BDNF (50 ng/ml) or conditional medium (1:1) were added to the medium. The migrated cells were visualized by crystal violet (0.05% w/v) staining. CellTiter-Glo Assay was used to quantify the migrated cells after 48 hours of incubation and removing the gel pieces.

**3D cell adhesion assay**

SphereCol human type I collagen coated beads (Advanced BioMatrix, San Diego, CA) suspended in 1ml cell growth medium (13.9 mg/ml, 6400 beads/ml with surface area of 5 cm^2^/ml) were mixed with 10^5^ cells. They were incubated on a rotator in the 37°C incubator for the desired time. After incubation, beads were washed with PBS three times to wash off the cells that did not adhere to the beads. CellTiter-Glo assay was performed to the beads to quantify the attached cells. A group with cells without beads was included for each time point to assess the total cell numbers. A negative control that contained only beads and medium was included for each time point to assess the background. The adhesion rate was calculated as [(adhesion-background)/(total-background)]%.

**Co-immunoprecipitation (co-IP)**

SureBeads (Bio-rad) were washed with PBS-T buffer (PBS + 0.1% Tween 20) three times and incubated with an antibody (1 µg) for 10 min at room temperature. Beads were washed with PBS-T buffer three times before the antibody-conjugated beads were ready for IP. Cell lysate was added to the beads and rotated for 1 hour at room temperature. Beads were magnetized to discard supernatant and thoroughly washed with PBS-T for 5 times. Laemmli buffer (40 µl) was added to the beads and incubated for 5 minutes at 90°C. Finally, beads were magnetized to move eluent to a new tube.

**Chromatin immunoprecipitation (CHIP)**

CHIP was performed as previously described (**Additional reference 2**). Quantitative PCR was performed to measure the levels of binding DNA. Primer sequences were listed in the supplemental information. Signals obtained from the IP CREB and IgG control samples were normalized to that of the input control samples. After normalization, signals of the IgG control groups were subtracted from the IP CREB groups. The fold of increase in CREB binding to TrkB promoter was obtained by comparing FT240-R175H cell group to the FT240 control group.

**TrkB recycling assay**

After serum starvation for 2 hours, FTE cells were trypsinized and washed with ice-cold PBS (pH=8.0). Three million cells were used for cell-surface protein biotinylation by incubating for 30 minutes with 0.3 mg/ml sulfo-NHS-S-S-biotin (ProteoChem, Hurricane, UT) at 4°C. Unreacted biotin was quenched with ice-cold Tris-buffered saline (TBS). Cells were washed with cold PBS and then incubated with 50 ng/ml BDNF in DMEM/F12 medium at 37°C for 30 minutes to induce TrkB internalization. Afterward, cells were cooled on ice. The remaining cell-surface biotin was removed by incubating in biotin-removal buffer for 15 minutes at 4°C (50 mM glutathione, 75mM NaCl, 75mM NaOH, 0.01 g/ml bovine serum albumin, 10mM EDTA). The internalized biotinylated receptors were protected from biotin removal. Subsequently, cells were rewarmed and incubated at 37°C in DMEM/F12 medium for 15 or 45 minutes to allow receptor recycling. A second round of biotin removal of biotinylated receptors was performed. At each step, a sample of 500,000 cells was lysed with 1x ELISA lysis buffer (Cell Signaling Technology, #9803).

In biotin-labelled TrkB ELISA, ultra-sensitive streptavidin coated 96-well plates (Eagle Biosciences, Amherst, NH) were used to capture the biotin-labelled proteins in cell lysate. Cell lysate (50 ul) was added to the well and incubated overnight at 4°C. The wells were washed with washing buffer (Boster Biological Technology, #AR1155) and incubated with blocking buffer (2% BSA in PBS). Anti-TrkB antibody was incubated in the wells for 1 hour at room temperature. After 5 times of washing, HRP-conjugated secondary antibody incubated in the wells for 1 hour at the room temperature. After 5 times of washing, SuperBrite ELISA HRP Chemiluminescence Substrate (BioVision, Milpitas, CA) was added to each well to read out the luminescence signals using a GloMax Microplate Luminometer (Promega). TrkB recycling rate is calculated using the ELISA luminescence signal data by subtracting the remaining biotin-labelled TrkB after the final biotin removal from the sample after recycling, and then normalizing to the amount of total internalized TrkB before recycling.

**Ex vivo adipose tissue adhesion**

Epididymal adipose tissues were dissected from nude mice. In each tube, one piece of 0.01 g adipose tissue was co-cultured with 100,000 RFP-labeled FTE cells in 0.5 ml DMEM/F12 medium in the presence or absence of 50ng/ml BDNF. The tubes were incubated on a rotator at 37°C to allow attachment of FTE cells to adipose tissues. After 24 hours incubation, the adipose tissue pieces were removed from the tubes. The tissues were examined under a fluorescence dissecting stereomicroscope to confirm the attachment of RFP-labelled FTE cells. The numbers of cells that were left in the suspension were counted and subtracted from the total cell numbers to calculate the numbers of cells that attached to the adipose tissues.

**Human Transcriptome array and IPA analysis**

FTE240 cells were treated with BDNF 50ng/ml in 3D culture for 24 hours. Untreated cells were cultured under the same condition as control. Three biological repeats were included in the experiment, in which three different batches of FTE240 cells were utilized. Each repeat included untreated control and BDNF-treated groups. RNA was extracted and analysed using Clariom D Human Transcriptome Array and GeneChip Scanner 3000 7G system (Thermo Fisher Scientific, Waltham, MA). Transcriptome Analysis Console software was used to analyse the array data. The RNA expression was compared between the untreated control and BDNF-treated groups. Data are deposited at Dryad Digital Repository (https://doi.org/10.5061/dryad.dv41ns1vd). Genes that were upregulated or downregulated in the BDNF-treated group than untreated control group over 2 folds with p<0.05 were used for Ingenuity Pathway Analysis (IPA).

**Statistics**

Numerical values are presented as mean±SD. For comparisons between two groups, P values were calculated using paired or unpaired two-tailed Student’s t-tests. One-way ANOVA was used to analyse more than two independent groups. Two-way ANOVA was used to compare the difference in experiments with two independent variables. The variance was similar between the groups that were compared. Sample sizes were determined by power calculation with 80% power at α=0.05. Data outliners were Identified using Grubbs’ test (GraphPad Prism). For in vivo experiments, the groups of mice were randomized with the consideration that each group had the same average body weight (with no statistical difference by student’s t-test). One investigator conducted the experiments and a different investigator who was blinded assessed the outcomes. In animal experiments, cage numbers and mouse ID numbers were used instead of treatment group names for blinding.
