# Supplementary material for: Ovarian BDNF promotes survival, migration, and attachment of tumor precursors originated from p53 mutant fallopian tube epithelial cells
Source: Oncogenesis. 2020 May 29;9(5):55. doi: 10.1038/s41389-020-0243-y (PMC7260207; doi:10.1038/s41389-020-0243-y)
Supplement: Supplementary file 2 — List of primer, antibodies, and drugs [file 41389_2020_243_MOESM2_ESM.pdf]

Summary: Primer sequences, antibody ordering information and dilution, and small molecule drugs

1) **Primer list:**

TrkB-sense	5'- TGAGGACCAGAAAGTTCGGC-3'
TrkB-sense	5'- GAGGCTCCAAAGGCACTTGA-3'
GAPDH-sense	5'- AATGAAGGGGTCATTGATGG-3'
GAPDH-antisense	5'-AAGGTGAA GGTCGGAGTCAA-3'
TrkB-sense (CHIP)	5'- TTGTCTGGAGGGTGTTATGGGT -3'
TrkB-sense (CHIP)	5'- TGCTACTGCTGGCGAAGTGA-3'
HTRA4-sense	5'-GTGATGTGATTGGCGTCAATTC-3'
HTRA4-antisense	5'-TGCCTAACTCGATCTGAAGGA-3'
CDK19-sense	5'-TCACCGTGCATCAAAAGCAAA-3'
CDK19-antisense	5'-TGGCAAAACCCATGTCAGCTA-3'
FAM3D-sense	5'-GCTGGGACAGAAGGCATTTGA-3'
FAM3D-antisense	5'- TCCCTGGATCGTCGTAGGAG-3'
MMP9-sense	5'-TTGGTCCACCTGGTTCAACT-3'
MMP9-antisense	5'-ACGACGTCTTCCAGTACCGA-3'
MMP2-sense	5'-TGATCTTGACCAGAATACCATCGA-3'
MMP2-antisense	5'-GGCTTGCGAGGGAAGAAGTT-3'
FN1-sense	5'-ACCTCGGTGTTGTAAGGTGG-3'
FN1-antisense	5'-CCATAAAGGGCAACCAAGAG-3'
ZEB1/2-sense	5'-TGCTCTAGAGGCTTCATTTGTCTT-3'
ZEB1/2-antisense	5'- CGCGGATCCATGGCGGATGGCCCC-3'
SLUG-sense	5'- TGACCTGTCTGCAAATGCTC -3'
SLUG-antisense	5'- CAGACCCTGGTTGCTTCAA -3'
SLITRK4-sense	5'-GAGCCTTCAATAAGCTCCACAA-3'
SLITRK4-antisense	5'-GGTCAAAGATGCGAATCGGAAA-3'
SNAIL-sense	5'-TCTGAGTGGGTCTGGAGGTG-3'
SNAIL-antisense	5'-CTCTAGGCCCTGGCTGCTAC-3'
TWIST1-sense	5'-GTCATGGCCAACGTGCGGGA-3'
TWIST1-antisense	5'-GCCGCCAGCTTGAG GGTCTG-3'
TRXR1-sense	5'-TCAGGGCCGTTCATTTTTAG-3'
TRXR1-antisense	5'-GATCTGCCCGTTGTGTTTG-3'
NQO1-sense	5'-GCCTCCTTCATGGCATAGTT-3'
NQO1-antisense	5'-GGACTGCACCAGAGCCAT-3'

GCS-sense	5'-CTTTCTCCCCAGACAGGACC-3'
GCS-antisense	5'-CAAGGACGTTCTCAAGTGGG-3'
AKR1C1-sense	5'-TTGACTTGCAGAAATCCAGC-3'
AKR1C1-antisense	5'-AAGCCAGGGCTCAAGTACAA-3'
HMOX1-sense	5'-GAGTGTAAGGACCCATCGGA-3'
HMOX1-antisense	5'-GCCAGCAACAAAGTGCAAG-3'
SOD1-sense	5'-CCACACCTTCACTGGTCCAT-3'
SOD1-antisense	5'-CTAGCGAGTTATGGCGACG-3'
SAIH3-sense	5'-CTGTGCATGTGTCCCTTGTTC-3'
SAIH3-antisense	5'-TGTCAACCCTATGGATCTGCC-3'

2) Antibody list:

**Target**	**Host species/ conjugation**	**Application**	**Dilution**	**Catalog number**	**Manufactures**
p53	Rabbit	Western blot, IF staining, co-IP	1:1000, 1:200, 1:100	18032	Cell Signaling Technology, Danvers, MA
TrkB	Mouse	Western blot, Flow cytometry, IF staining, co-IP	1:200, 1:100, 1:200, 1:200	sc-377218	Santa Cruz Biotechnology, Dallas, TX
TrkB	Rabbit	Western blot	1:250	bs-0175R-TR	Bioss Antibodies, Woburn, MA
GGA3	Rabbit	Western blot	1:1000	8027	Cell Signaling Technology
p-TrkB	Mouse	Western blot	1:250	sc-8058	Santa Cruz Biotechnology
AKT	Rabbit	Western blot	1:1000	2920	Cell Signaling Technology
p-AKT	Rabbit	Western blot	1:1000	4060	Cell Signaling Technology
ERK	Rabbit	Western blot	1:1000	4695	Cell Signaling Technology
p-ERK	Rabbit	Western blot	1:1000	9160	Cell Signaling Technology
Tubulin	Mouse/HRP	Western blot	1:10000	HRP-66031	Proteintech, Rosemont, IL, USA
GAPDH	Mouse/HRP	Western blot	1:10000	HRP-60004	Proteintech
mouse IgG	Goat/HRP	Western blot, ELISA	1:1000, 1:5000	SA00001-1	Proteintech

rabbit IgG	Goat/HRP	Western blot	1:10000	SA00001-2	Proteintech
mouse IgG	Goat/Alexa Flour 488	Flow cytometry, IF staining	1:1000	8890	Cell Signaling Technology
Cytokeratin 8	Rabbit	IF staining	1:200	10384-AP	Proteintech
GGA3	Rabbit	IF staining	1:200	612310	BD Transduction laboratories, San Jose, CA
rabbit IgG	Alexa Fluor 594	IF staining	1:1000	8889	Cell Signaling Technology
CREB		Western blot, CHIP	1:1000, 1:250	9197	Cell Signaling Technology
GGA3	Mouse	Co-IP	1:200	sc-135923	Santa Cruz Biotechnology
Isotype control	Mouse	Co-IP control	1:250	3900	Cell signalling Technology
Isotype control	Rabbit	Co-IP control	1:250	61656	Cell signalling Technology
TrkB	Mouse	ELISA	1:200	A15019C	Biolegend

3) Drug list:

**Drug name**	**Chemical name**	**Catalog number**	**Company**	**Preparation**
ANA-12	N-[2-[[(Hexahydro-2-oxo-1H-azepin-3-yl)amino]carbonyl] phenyl]-benzo[b]thiophene-2-carboxamide	B5712	APEXBIO	Dissolve in DMSO to make10 mM stock.
BDNF	Brain derived neutrophic factor	3897	Cell Signaling Technology	Reconstitute with sterile water to make 0.1 mg/ml stock.
BDNF	Brain derived neutrophic factor	CYT-207	Peptides International	Reconstitute with sterile water to make 0.1 mg/ml stock.
DAPI	4',6-Diamidino-2-Phenylindole, Dihydrochloride	D1306	Thermo Fisher	Dissolve in deionized water to make a 5 mg/mL stock solution.
sulfo-NHS-S-S-biotin	Sulfosuccinimidyl-2-(biotinamido)-ethyl-1,3'-dithiopropionate	b2104	ProteoChem	Make a 6 mg/mL stock solution (~10mM) in water immediately before use.
