# Supplementary material for: Ovarian BDNF promotes survival, migration, and attachment of tumor precursors originated from p53 mutant fallopian tube epithelial cells
Source: Oncogenesis. 2020 May 29;9(5):55. doi: 10.1038/s41389-020-0243-y (PMC7260207; doi:10.1038/s41389-020-0243-y)
Supplement: Supplementary file 3 — Supplementary figures [file 41389_2020_243_MOESM3_ESM.pdf]

Supplemental Figures

Description: This section contains references and figures that are directly relevant to the conclusion of an article but cannot be included in the printed version owing to space constraints.

Additional References

1. Uhlen M, Fagerberg L, Hallstrom BM, Lindskog C, Oksvold P, Mardinoglu A, et al. Proteomics. Tissue-based map of the human proteome. Science 2015;347(6220):1260419.
2. Nelson JD, Denisenko O, Bomsztyk K. Protocol for the fast chromatin immunoprecipitation (ChIP) method. Nat Protoc 2006;1(1):179-85.

Supplemental Figures

S1

Normal fallopian tube (age 46) **Normal fallopian tube (age 34)**

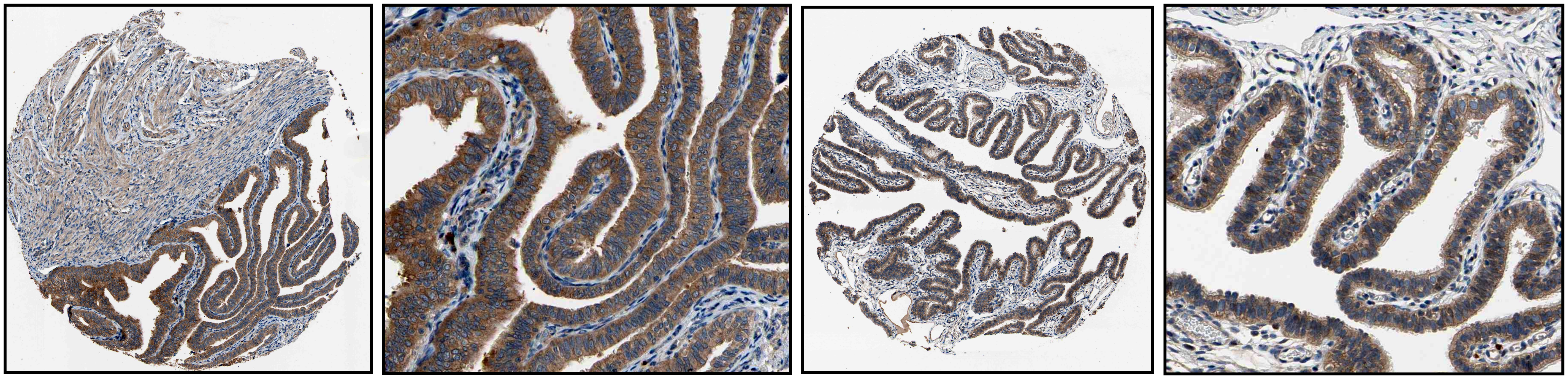

Figure S1. **The expression of TrkB in human fallopian tubes.** TrkB was detected in the cellular membrane and cytoplasm of normal human fallopian tube epithelium (cited Human Protein Atlas version 18.1, **Additional reference 1**).

S2

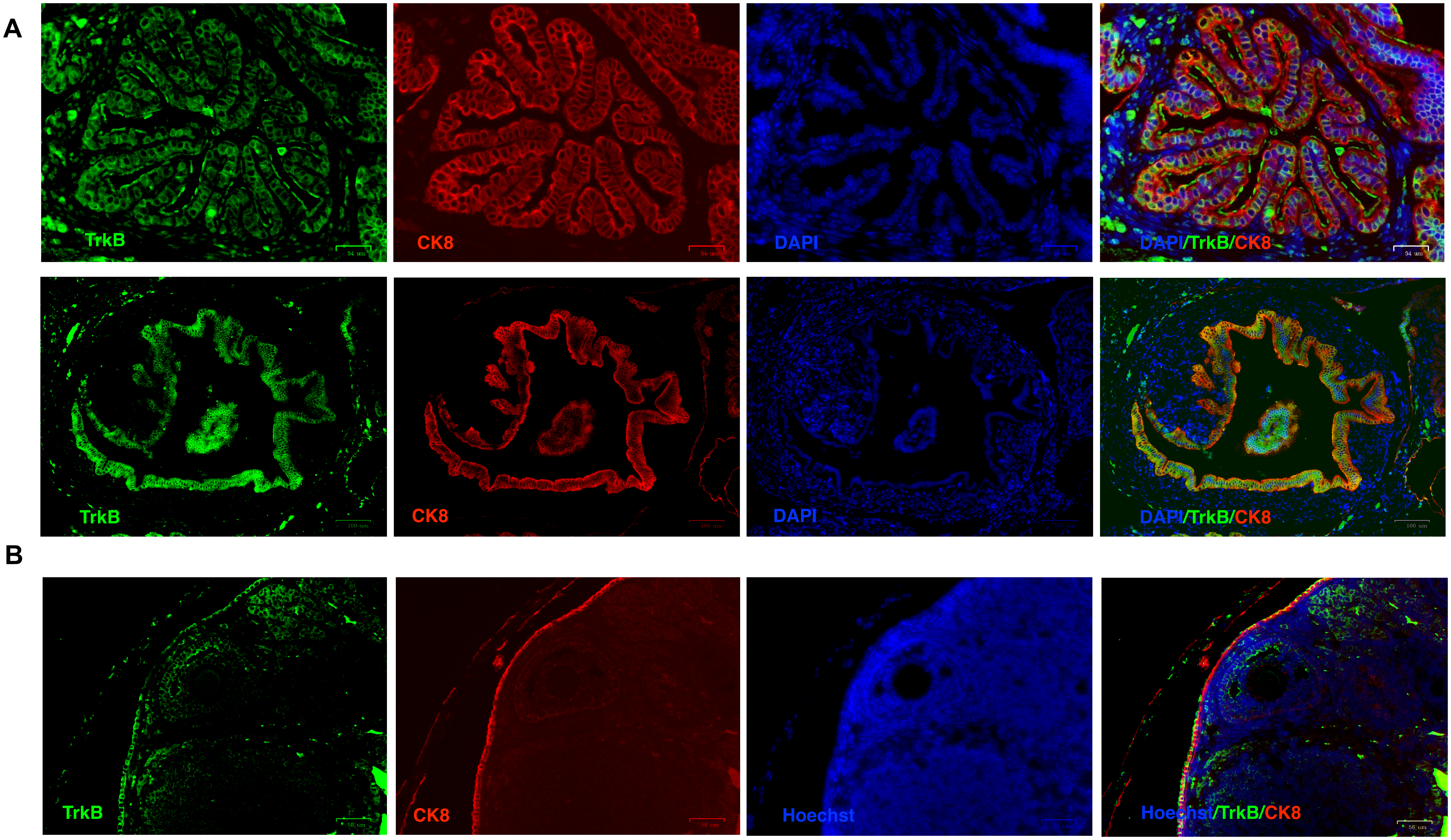

Figure S2. **The expression of TrkB mouse in ovidcuct and ovary.** For the immunofluorescence (IF) staining of TrkB (green) and cytokeratin 8 (CK8, red) in mouse ovary, DAPI was used to stain nuclear DNA (blue). In mouse oviduct TrkB was expressed in the membrane of oviduct epithelial cells and overlapped with CK8, an epithelial cell marker (S2A). TrkB was also detected in the mouse OSEs (S2B). FTEs and OSEs are both hypothesized to be the cells of origin of HGSOC.

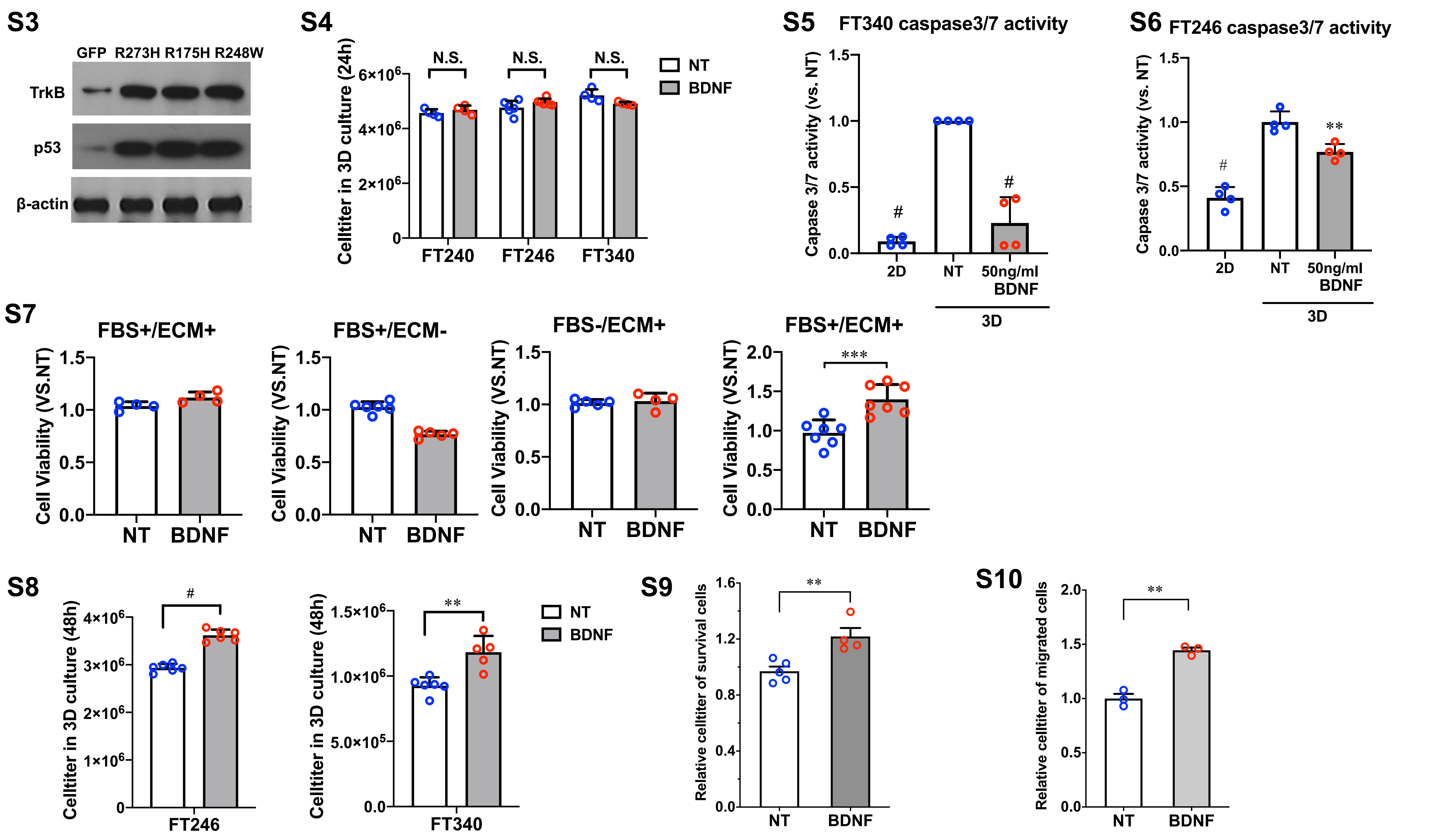

Figure S3. Representative images of western blot of FT246 cells. The whole cell lysate of FTE246 cells overexpressing green fluorescent protein (GFP), p53R273H, p53R175H, or p53R248W were analyzed.

Figure S4. Cell viability of FTE cells after 24 hours in 3D culture.
BDNF (50ng/ml) did not change the numbers of viable three FTE cell lines after 24 hours of 3D culture. n=3. N.S., not significant for unpaired student's t-test.

Figure S5. Caspse3/7 activity of FT340 cells at 24 hour.
BDNF suppressed Caspase3/7 activity of FT340 cells in 3D cluture. Data were normalized to Caspase3/7 activity of cells in 2D culture. n=3. #p<0.0001 for one-way ANOVA followed by Tukey's HSD test.

Figure S6. Caspse3/7 activity of FT246 cells at 24 hour.
BDNF (50ng/ml) suppressed Caspase3/7 activity of FT246 cells in 3D cluture. Data were normalized to Caspase3/7 activity of cells in 2D culture. n=4. *p<0.05 for unpaired student's t-test.

Figure S7. The effects of BDNF treatment on the viability of FTEs in different conditions after 48 hours. The viability of human FTE cell line FT240 under 4 different conditions when they treated or untreated (NT) with 50 ng/ml BDNF for 48 hours. *p<0.05 for unpaired student's t-test. FBS, fetal bovine serum. ECM, extracellular matrix. **Note:** Only in the absence of FBS and ECM did FTEs respond to BDNF.

Figure S8. The effects of BDNF treatment on the viability of FTEs in 3D culture after 48 hours. The viability of human FTE cell lines FT246 and FT340 under 3D no FBS condition when they treated or untreated (NT) with 50 ng/ml BDNF for 48 hours. *p<0.05 for unpaired student's t-test.

Figure S9.

Figure S10.

Supplemental Figures

S11

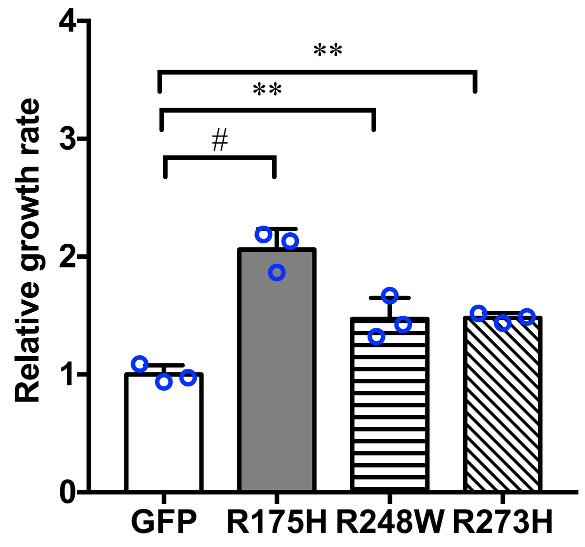

S12

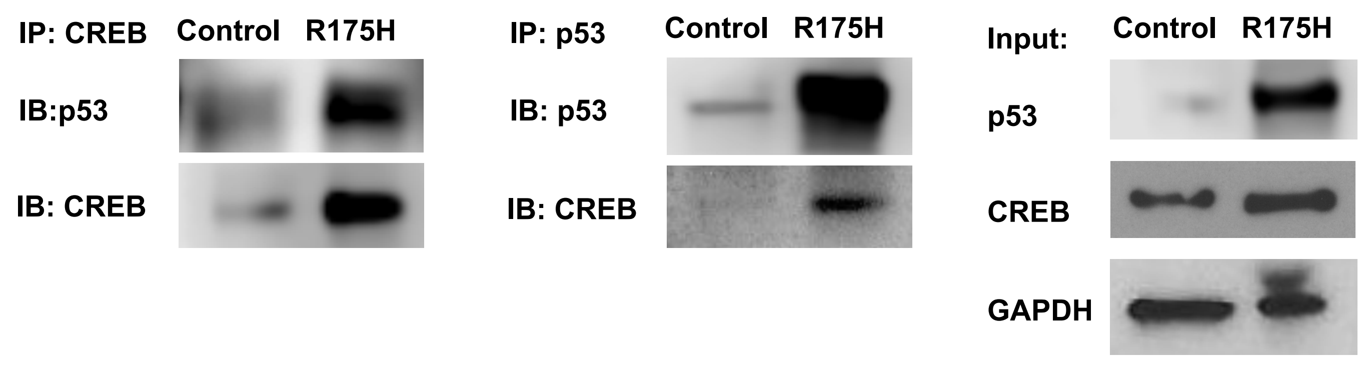

S13

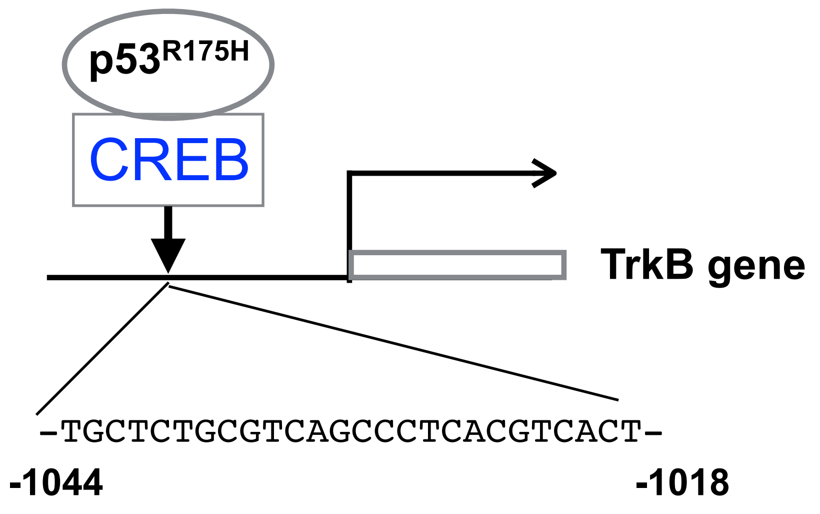

S14

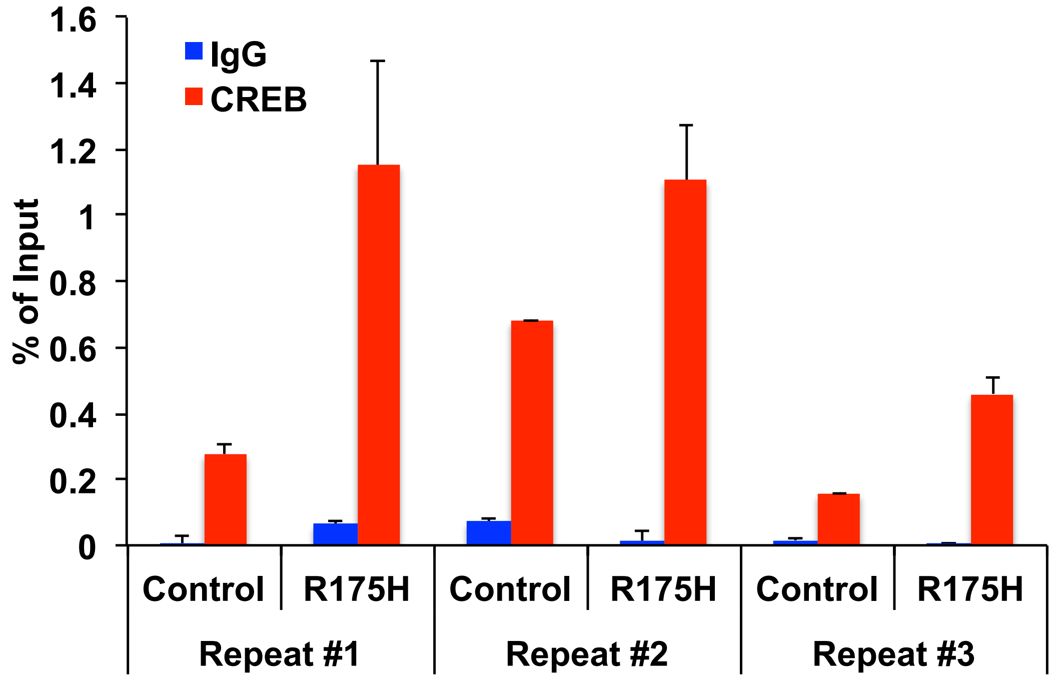

Figure S11. Growth rate of FTE cells.
FT240 cells expressing mutant p53 R175H, R248W, and R273H proliferated faster than the control FT240 cells expressing GFP in 2D culture. n=3. The same numbers of cells were plated on day 0. After 48 hours, the relative cell growth rates were calculated by comparing the luminescence-based celltiter to the control cells. **p<0.001, #p<0.00001, for one-way ANOVA followed by Tukey's HSD test.

Figure S12. Co-immunoprecipitation (co-IP) of p53 and CREB in FT240 cells (Method as described in **Additional reference 2**).

Figure S13. Schematic diagram of the CREB binding site in TrkB gene (**NTRK2).**

Figure S14. Chromatin-IP of CREB followed by quantification of the CREB-binding DNA fragment in TrkB gene. The experiment was repeated 3 times. Signals obtained from the IP-CREB and IP-IgG control samples were normalized to that of the input control samples. After the normalization, signals of the IgG control groups were subtracted from the IP-CREB groups. The fold of increase in CREB binding to TrkB promoter was obtained by comparing FT240-R175H cell group to the FT240 control group and presented in Figure 3E.

Supplemental Figures

S15

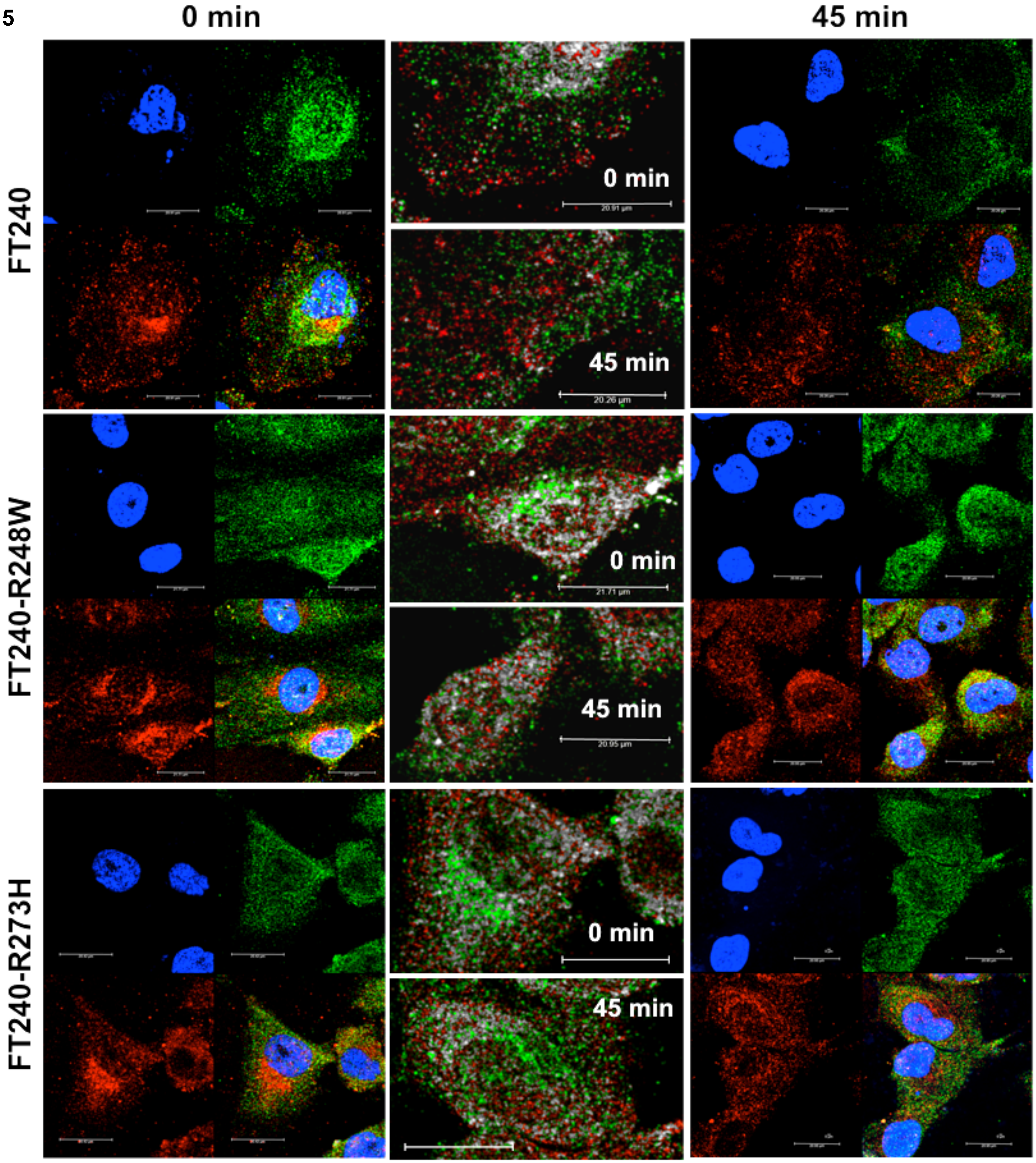

Figure S15. Large size images for Figure 4F

Mutant p53 is associated with higher levels of TrkB-GGA3 colocalization in FTE cells. Cells were treated with 50ng/ml BDNF for 0-45 minutes. IF staining of TrkB (green) and GGA3 (red) indicated colocalization. The colocalized TrkB and GGA3 signals were labeled as white in the middle panel. DAPI was used to stain nuclear DNA (blue).

Supplemental Figures

S16

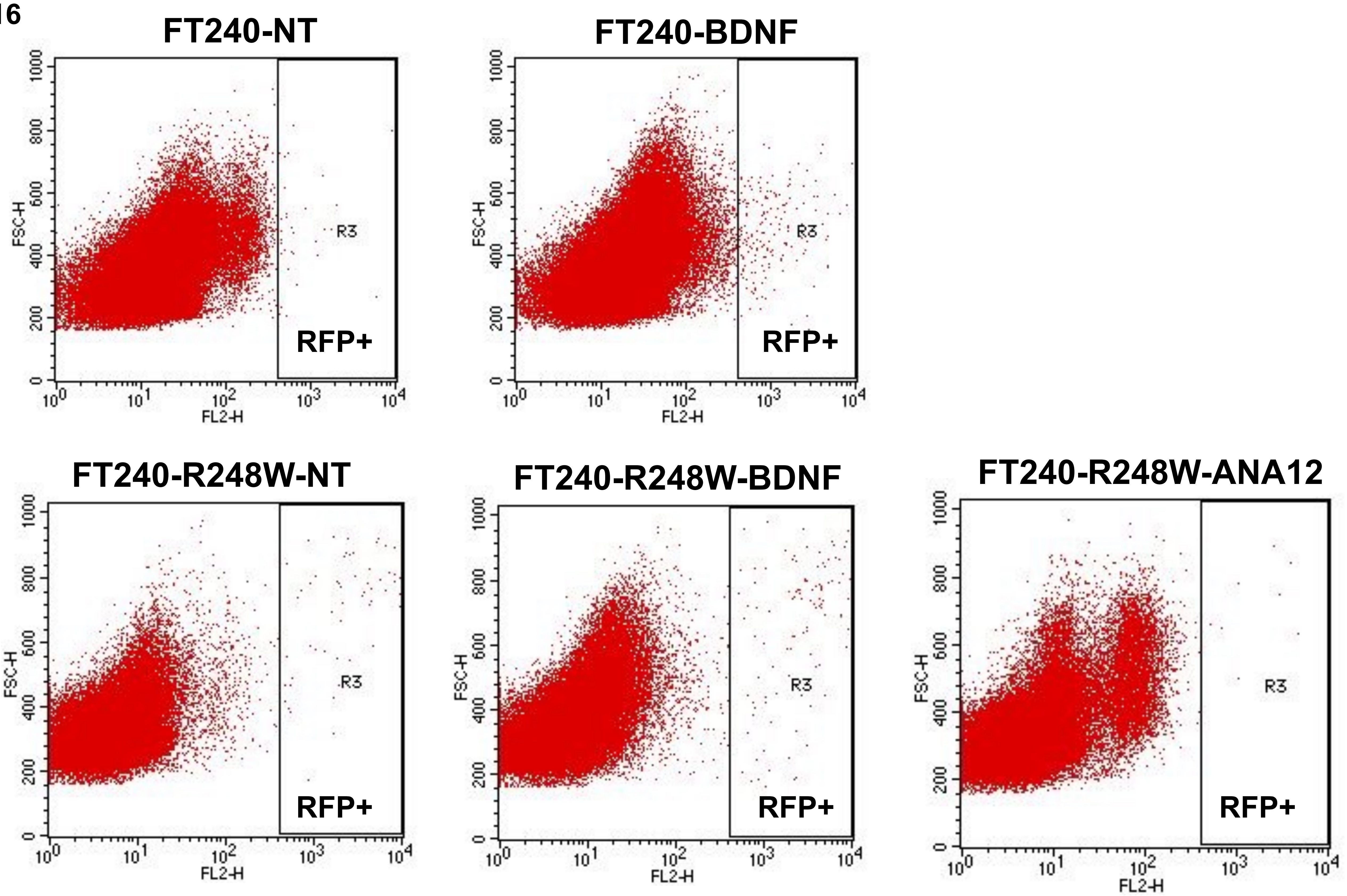

Figure S16. Represetntive flowcytometry graphs for detection of RFP+ FTE cells in the perinoneal cells.

S17

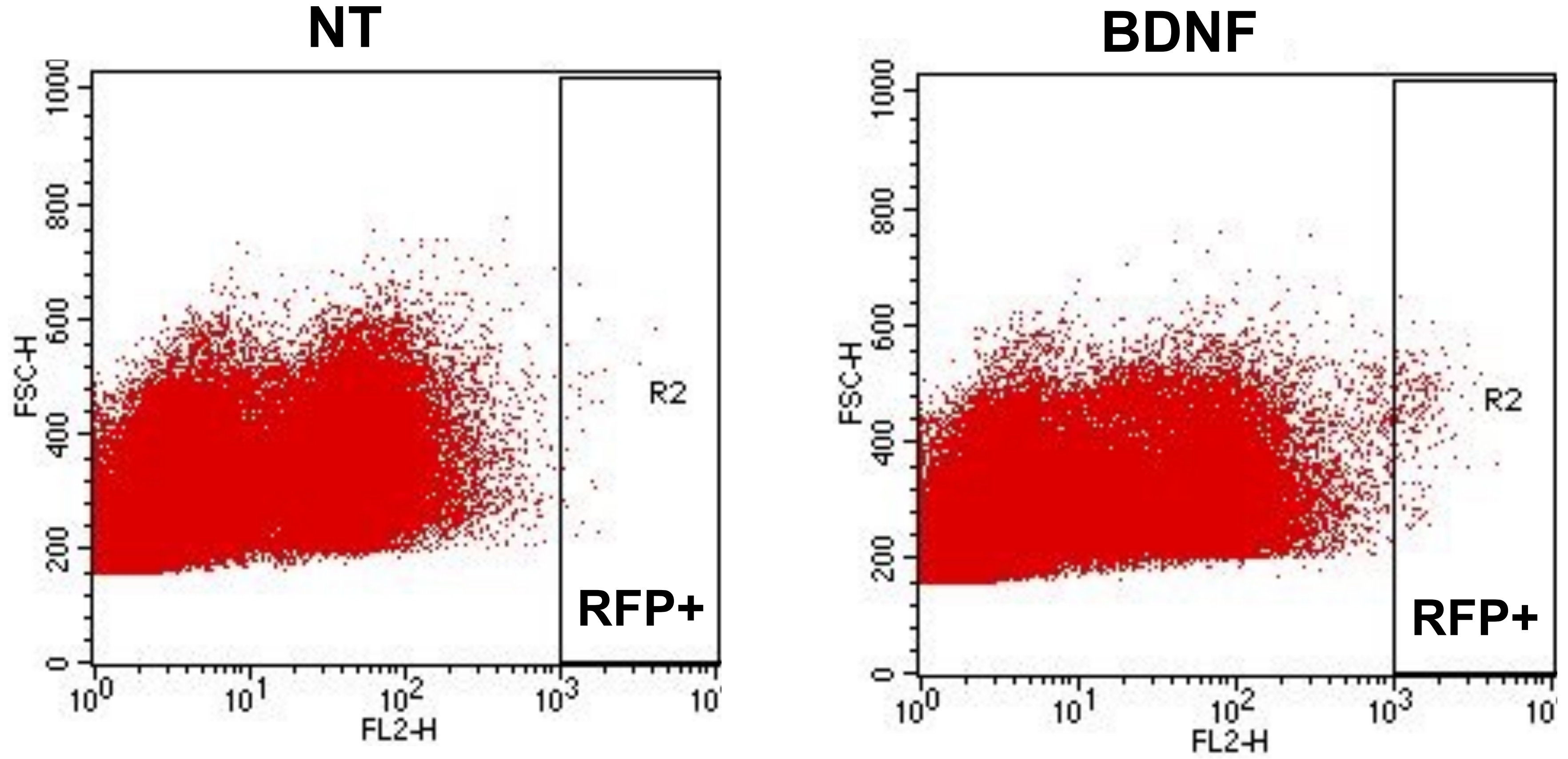

Figure S17. Represetntive flowcytometry graphs for detection of RFP+ OVC cells in the perinoneal cells.
